# Nitrogen Vacancy Rich Tubular g-C_3_N_4_ Modified with FePc for Efficient and Reusable Photo-Fenton Degradation of Oxytetracycline

**DOI:** 10.3390/molecules31183229

**Published:** 2026-09-12

**Authors:** Xinyi Yang, Yuxin Tang, Fei Qi, Zhihan Xue, Huiying Zhang, Zhaohai Ni, Bo Feng, Ziyang Yue, Guangbo Che

**Affiliations:** 1 College of Safety Science and Engineering, Liaoning Technical University, Huludao 125105, China; 2 Jilin Provincial Key Laboratory of Western Jilin’s Clean Energy, Baicheng Normal University, Baicheng 137000, China; 3 College of Chemistry, Changchun Normal University, Changchun 130032, China; 4 College of Science, Liaoning Technical University, Fuxin 123000, China

**Keywords:** tubular g-C_3_N_4_, nitrogen vacancies, iron phthalocyanine, photo-Fenton

## Abstract

Efficient and reusable photo-Fenton catalysts require the rational integration of photocatalytic platforms, effective H_2_O_2_ activation sites, and practical immobilization strategies. Herein, nitrogen-vacancy-rich tubular g-C_3_N_4_ (HCNT) was prepared as the photocatalytic platform. Iron (II) phthalocyanine (FePc) was then loaded onto HCNT to form the FePc/HCNT composite for antibiotic degradation. The FePc/HCNT composite was further immobilized in a poly (vinylidene fluoride) (PVDF) membrane, denoted as FePc/HCNT-PVDF, to facilitate catalyst recovery and reuse. Benefiting from the micrometer-scale tubular morphology, the FePc/HCNT remained exposed on the membrane surface, preserving accessible catalytic interfaces and reducing encapsulation within the polymer matrix. The tubular architecture facilitated reactant transport, while surface nitrogen vacancies enhanced photogenerated-carrier separation and utilization. Component-dependent experiments revealed that FePc served as the primary center for H_2_O_2_ activation, whereas HCNT functioned as a defect-engineered tubular photocatalytic platform that promoted photogenerated-carrier separation and reactive oxygen species formation. The optimized FePc/HCNT achieved 94% oxytetracycline degradation within 60 min, with an apparent rate constant of 0.04462 min^−1^, approximately 11 times higher than bulk g-C_3_N_4_, and exhibited broad-spectrum degradation capability toward various organic pollutants, including rhodamine B, tetracycline, amoxicillin, and ciprofloxacin. The FePc/HCNT-PVDF membrane removed 90% of oxytetracycline within 60 min and maintained stable performance over ten cycles. This work provides insights into the integrated roles of nitrogen vacancy regulation, FePc-mediated H_2_O_2_ activation, and morphology-assisted membrane immobilization in the development of reusable g-C_3_N_4_-based photo-Fenton catalysts.

## 1. Introduction

Antibiotic residues are frequently detected in wastewater and natural aquatic environments and have become contaminants of increasing concern because of their potential environmental risks [1]. Tetracycline antibiotics, including oxytetracycline (OTC), are representative aquatic micropollutants and have been widely detected in water environments [2]. Although adsorption and membrane filtration can remove antibiotics from water, adsorption mainly transfers the pollutants to a solid phase without destroying their molecular structures, while membrane filtration may involve relatively high operating costs and further treatment of the concentrated pollutants. Advanced oxidation processes (AOPs) capable of generating reactive oxygen species (ROS) have therefore been widely investigated for the degradation of antibiotic contaminants, and photo-Fenton treatment has demonstrated effective antibiotic removal even at the pilot scale [3]. Among these technologies, Fenton technology has attracted particular attention due to its high efficiency, relatively low cost, and ease of industrial application. However, the conventional homogeneous Fe^2+^ catalyzed Fenton process only works efficiently under strongly acidic conditions (pH ≈ 3) and generates large amounts of iron-containing sludge due to the precipitation of Fe^3+^ as iron hydroxide, causing secondary pollution [4]. To reduce sludge production and widen the applicable pH range, researchers have developed heterogeneous Fenton systems using immobilized Fe(II) catalysts. Iron-based solid catalysts used in such systems include nano zero-valent iron, Fe_2_O_3_ [5], Fe_3_O_4_ [6], and FeS_2_ [7]. However, the reduction of Fe(III) species in heterogeneous catalysts is more difficult than that of free Fe^3+^ in solution, and the regeneration of Fe(II) largely depends on electron donation from H_2_O_2_, which lowers the utilization efficiency of H_2_O_2_. Introducing external electrons into the heterogeneous Fenton system has proven effective in accelerating Fe(II) regeneration and improving H_2_O_2_ utilization. Since photocatalytic processes can continuously supply photogenerated electrons, coupling photocatalysis with heterogeneous Fenton reactions enables fast Fe(II)/Fe(III) cycling driven by these electrons [8]. Thus, heterogeneous photo-Fenton systems are expected to overcome the drawbacks of conventional heterogeneous Fenton processes and further improve catalytic efficiency [9].

Organic phthalocyanine ligands can stabilize iron ions and form atomically dispersed Fe(II) sites. Iron(II) phthalocyanine (FePc) has a planar conjugated macrocyclic structure, exhibits good semiconducting and photoconductive properties, and shows strong absorption in the visible region. In addition, FePc is low-cost and readily available, making it suitable for large-scale preparation. However, FePc tends to aggregate in aqueous solutions due to poor dispersibility, leading to reduced catalytic performance. Excessive aggregation also shields the iron centers, making them susceptible to decomposition by oxidizing species and thereby lowering stability [10]. Therefore, a suitable support is needed to disperse FePc and prevent its aggregation.

Graphitic carbon nitride (g-C_3_N_4_) has emerged as an attractive metal-free photocatalyst due to its abundant raw materials, stable physicochemical properties, tunable structure, simple synthesis, and environmental friendliness [11,12,13,14]. Nevertheless, pristine g-C_3_N_4_ suffers from a low density of exposed active sites and a high recombination rate of photogenerated charge carriers, resulting in limited photocatalytic activity [15]. Various modification strategies have been explored, including vacancy engineering [16], elemental doping [17], heterojunction construction [18], and morphology design [19]. Among these, synergistic engineering of morphology and vacancies is an effective route to enhance the activity of g-C_3_N_4_. Compared with two-dimensional nanosheets, three-dimensional g-C_3_N_4_ tubes prepared by supramolecular self-assembly exhibit stronger light scattering, quantum confinement effects, and oriented electron transport channels, thus showing better photocatalytic performance [20]. However, thick tube walls can hinder the diffusion of photogenerated carriers and the exposure of active sites [21]. Ultrathin porous g-C_3_N_4_ tubes are attractive because they provide higher specific surface area, more abundant active centers, and shorter diffusion paths.

In addition to morphology control, defect engineering, particularly the introduction of nitrogen vacancies (N_v_s), has been recognized as an effective approach to modulate the electronic structure of g-C_3_N_4_. Nitrogen vacancies can create mid-gap states, extend light absorption, and serve as active sites for molecular oxygen activation. Nevertheless, the photocatalytic activity of vacancy-engineered g-C_3_N_4_ is still limited by the sluggish kinetics of surface redox reactions. Constructing FePc-based molecular heterojunctions on defect-engineered g-C_3_N_4_ provides an opportunity to integrate semiconductor photocatalysis with molecular Fenton catalysis [22]. Nevertheless, several key issues remain unresolved: (i) how surface defects on g-C_3_N_4_ influence the catalytic behavior of FePc-based heterojunctions; (ii) the respective roles of FePc molecular sites and semiconductor components during H_2_O_2_ activation and ROS generation; and (iii) how to achieve efficient and reusable photo-Fenton catalysts with suppressed metal leaching [23,24].

In this work, nitrogen vacancy rich tubular g-C_3_N_4_ was modified with iron (II) phthalocyanine (FePc/HCNT) to construct a reusable photo-Fenton catalyst for antibiotic degradation. The tubular g-C_3_N_4_ substrate provides a favorable photocatalytic framework, while surface N_v_s regulate the surface chemical environment and contribute to improved catalytic performance. The anchored FePc molecular centers serve as efficient active sites for H_2_O_2_ activation. Furthermore, benefiting from the micrometer-sized tubular architecture of tubular g-C_3_N_4_, the FePc/HCNT catalyst can be effectively immobilized onto a PVDF membrane while maintaining sufficient exposure and accessibility of catalytic surfaces, avoiding the shielding of active sites caused by conventional membrane encapsulation. The resulting catalytic membrane system exhibits excellent recyclability and operational stability during repeated degradation cycles, demonstrating great potential for practical wastewater treatment applications. Overall, this work demonstrates the combined effects of tubular structural engineering, surface nitrogen vacancy regulation, molecular FePc functionalization, and membrane immobilization on the photo-Fenton behavior of g-C_3_N_4_-based materials. These findings provide a fundamental basis for understanding and further designing immobilized g-C_3_N_4_-based photo-Fenton systems.

## 2. Experimental

### 2.1. Chemicals and Materials

Melamine (C_3_H_6_N_6_, 99%), oxytetracycline (C_22_H_24_N_2_O_9_, ≥98%), iron phthalocyanine (C_32_H_16_FeN_8_, FePc, 98%), ethanol (C_2_H_6_O, ≥99.7%), hydrochloric acid (HCl, 35%), sodium hydroxide (NaOH, 98%), sodium sulfate (Na_2_SO_4_, 98%), AgNO_3_ (99.8%), (NH_4_)_2_C_2_O_4_ (99.8%), ethylenediaminetetraacetic acid (EDTA, 99%), Tert-butyl alcohol (TBA, 99%), L-histidine (≥99%), 5,5-dimethyl-1-pyrroline N-oxide (DMPO, 97%), 2,2,6,6-tetramethyl-4-piperidone hydrochloride (TEMP, 97%), 1-Methyl-2-pyrrolidinone (NMP) and Poly Vinylidene Fluoride (PVDF) were purchased from Aladdin Scientific Corp. (Shanghai, China) or Macklin Inc. (Shanghai, China). All chemicals were used as received without further purification. Deionized water was used to prepare all solutions.

### 2.2. Synthesis of Photocatalysts

A total of 1 g of melamine was completely dissolved in 70 mL of water at 85 °C. The resulting solution was transferred into a Teflon-lined autoclave and heated at 180 °C for 12 h. After naturally cooling to room temperature, the precipitate was collected, washed with water, and dried at 60 °C to obtain a precursor powder. The powder was then calcined in a tube furnace under an Ar flow. The temperature was raised to 550 °C at a heating rate of 2.3 °C min^−1^ and held at that temperature for 4 h. After cooling to room temperature, a yellow powder was obtained, which was tubular g-C_3_N_4_ and denoted as CNT.

A total of 0.5 g of CNT powder was placed in a quartz crucible and positioned at the center of a tube furnace. The sample was heated to 550 °C under a H_2_/Ar atmosphere (5:95 *v*/*v*) for 120 min. After cooling to room temperature, a yellow powder was obtained, which was g-C_3_N_4_ tubes with surface nitrogen vacancies and denoted as HCNT.

A total of 0.2 g of HCNT was dispersed in 20 mL of ethanol by ultrasound (40 Hz) and stirred continuously at 60 °C for 60 min to form a suspension. A certain amount of FePc was dissolved in 40 mL of anhydrous ethanol, and the solution was added dropwise into the HCNT suspension. The mixture was stirred at 60 °C for another 60 min. After naturally cooling to room temperature, the product was washed several times with deionized water and finally dried in an oven at 60 °C overnight. FePc/HCNT-X samples were prepared by varying the FePc loading amount (3, 5, 10, 20, and 30 mg), where X represents the mass of FePc added.

For comparison, bulk g-C_3_N_4_ (CN) was prepared by calcining melamine in a muffle furnace at 5 °C min^−1^ for 4 h. Sheet-like g-C_3_N_4_ (CNS) was then obtained by dispersing CN in ethanol and ultrasonicating for 5 h. Meanwhile, the FePc + HCNT mixture was prepared by physically grinding a certain amount of FePc together with the HCNT sample.

### 2.3. Preparation of the FePc/HCNT-PVDF Membrane

FePc/HCNT-PVDF membranes were prepared by the following method. PVDF (1.8 g) was dissolved in NMP (12 mL) at 60 °C under stirring (350 rpm). After 6 h of stirring, a homogeneous milky white solution formed. FePc/HCNT powder was then added to reach a PVDF mass fraction of 5%, and stirring continued for another 10 h. The suspension was cast onto a glass plate, which was then immersed in deionized water at room temperature to induce coagulation. The membrane was peeled off and washed with deionized water to remove residual NMP. A pristine PVDF membrane was also prepared under the same conditions without adding FePc/HCNT.

### 2.4. Characterizations

Powder X-ray diffraction (XRD) patterns were recorded on a Panalytical X’Pert3 diffractometer (Malvern Panalytical, Almelo, The Netherlands). Sample morphologies were examined using scanning electron microscopy (SEM, Hitachi SU8010, Tokyo, Japan) and transmission electron microscopy (TEM, FEI Tecnai F20, Thermo Fisher Scientific, Hillsboro, OR, USA). X-ray photoelectron spectroscopy (XPS, ESCALAB 250Xi, Thermo Fisher Scientific, Waltham, MA, USA) with Al Kα radiation was used to analyze chemical states and valence band (VB) edges of the photocatalysts. UV–vis diffuse reflection spectra were obtained on a Hitachi U-3900 spectrometer (Tokyo, Japan). Surface topography was imaged using an atomic force microscope (AFM, Bruker MultiMode 8, Bruker Nano Inc., Santa Barbara, California, USA). Specific surface areas were determined with a Brunauer–Emmett–Teller (BET) surface area analyzer (Micromeritics ASAP 2020 Plus, Norcross, GA, USA). Photoelectrochemical measurements were carried out on a CHI660E electrochemical station (Chenhua, Shanghai, China) equipped with a 300 W xenon lamp. A three-electrode system was used, consisting of a sample-coated ITO conductive glass as the working electrode, Ag/AgCl as the reference electrode, a platinum wire as the counter electrode, and 0.25 mol/L Na_2_SO_4_ solution as the electrolyte. Reactive radicals generated in the photocatalytic reaction were analyzed by electron spin resonance (ESR, A300, Bruker BioSpin, Rheinstetten, Germany). Fourier transform infrared (FT-IR) spectra were recorded on a Tensor 27 spectrometer (Bruker, Billerica, MA, USA) in the range of 400–4000 cm^−1^. Photoluminescence (PL) spectra were measured on a Hitachi F7100 spectrophotometer. A liquid chromatography–mass spectrometry (LC-MS, Agilent 1290II-6460, Santa Clara, CA, USA) was used to identify the intermediates generated during photocatalytic degradation of OTC. The mineralization efficiency was evaluated by measuring total organic carbon (TOC) with a Shimadzu TOC-L-CPH (Shimadzu, Kyoto, Japan) analyzer in the TC-IC mode (indirect method). The separation was performed on a Waters BEH-C18 column (2.1 × 100 mm, 2.5 μm) at 25 °C. The mobile phase consisted of methanol (A) and 0.1% formic acid in water (B), with a flow rate of 0.2 mL min^−1^. The initial ratio of A to B was 5:95 (*v*/*v*). The gradient program was set as follows: from 5% A at 0 min to 70% A at 9 min, held at 70% A for 3 min, then returned to 5% A at 12 min and held for another 5 min. Mass spectrometry was operated in positive ion mode under the following conditions: capillary voltage 3200 V, atomization gas flow rate 100 L min^−1^, ion source temperature 300 °C, and *m*/*z* range of 50–600. The injection volume was 5 μL.

### 2.5. Measurement of Photo-Fenton Activity

Photocatalytic degradation of OTC was carried out in a CEL-PCRD300-12 photoreactor (Beijing Huajiao, Beijing, China). A total of 20 mg of catalyst was dispersed into 30 mL of OTC solution at a given initial concentration. The suspension was stirred in the dark at 1500 r⋅min^−1^ for 30 min to reach adsorption–desorption equilibrium. Subsequently, 0.5 mL of H_2_O_2_ solution (3 wt.%) was introduced into the suspension, resulting in an initial H_2_O_2_ concentration of 15 mmol L^−1^ in the reaction system. The suspension was then exposed to white LED light (132 mW cm^−2^) under continuous stirring. At 10 min intervals, 2 mL aliquots were taken and filtered through a 0.45 μm syringe filter to remove catalyst particles. The residual OTC concentration in the filtrate was measured using a UV–vis spectrophotometer (Genesys 30, Thermo Fisher Scientific, Madison, WI, USA) at 268 nm. The OTC concentration was calculated by Equation (1), and the apparent rate constant was derived from pseudo-first-order kinetics using Equation (2), where C_0_ and C are the OTC concentrations at initial time and time t, respectively. The apparent first-order rate constant (k, min^−1^) is the slope of the degradation kinetic plots.

(1) $$\mathrm{TC}\left(\%\right)=C/C_{0}\times 100\%$$

(2) $$\ln\;(C/C_{0})=-\mathrm{kt}$$

The catalytic performance of the FePc/HCNT-PVDF membrane was tested in a water-jacketed beaker to keep the reaction at room temperature. A 300 W Xe lamp served as the light source (Figure S1). A membrane piece of 4 × 4 cm^2^ (containing about 0.002 g of FePc/HCNT powder) was used for photo-Fenton degradation of OTC.

### 2.6. Determination of H_2_O_2_ Consumption During the Photo-Fenton Process

The residual concentration of H_2_O_2_ during the photocatalytic reaction was determined by the iodometric method. Under acidic conditions (pH = 4), H_2_O_2_ oxidizes iodide ions (I^−^) to generate triiodide ions (I_3_^−^), which exhibit a characteristic absorption peak at 352 nm. The absorbance intensity was recorded using a UV–vis spectrophotometer, and the residual H_2_O_2_ concentration was calculated based on a calibration curve established with standard H_2_O_2_ solutions. Specifically, 0.09 mmol of H_2_O_2_ was added into the reaction system under dark conditions, followed by visible light irradiation for 60 min. After the reaction, 1 mL of the suspension was collected, filtered, and diluted with 19 mL of ultrapure water (20-fold dilution). Subsequently, 2 mL of the diluted solution was transferred into a quartz cuvette, followed by sequential addition of 1 mL potassium iodide (KI) solution (0.4 mol L^−1^) and 1 mL potassium hydrogen phthalate buffer solution (0.1 mol L^−1^, pH = 4). The mixture was allowed to react in the dark for 30 min to ensure complete color development. Finally, the absorbance at 352 nm was measured, and the residual H_2_O_2_ concentration was determined using the established calibration curve.

### 2.7. Detection of Hydroxyl Radicals During the Photo-Fenton Process

To verify the involvement of hydroxyl radicals (•OH) during the photo-Fenton degradation process, terephthalic acid (TA) was employed as a fluorescent probe to monitor the time-dependent generation of •OH radicals by photoluminescence (PL) spectroscopy. Specifically, 10 mg of catalyst was dispersed in 25 mL of deionized water by ultrasonication for 10 min, denoted as solution A. Meanwhile, 0.1 mmol of disodium terephthalate (C_8_H_4_Na_2_O_4_) was dissolved in 25 mL of deionized water to obtain solution B. Subsequently, solution B was added into solution A and mixed thoroughly. A 6 mL aliquot of the mixture was collected as the dark control sample without light irradiation, while the remaining suspension was subjected to visible light irradiation. Aliquots were collected after 30 and 60 min of irradiation, respectively, and their PL spectra were recorded to evaluate the generation of •OH radicals.

## 3. Results and Discussion

### 3.1. Synthesis and Characterization

**Scheme 1:**
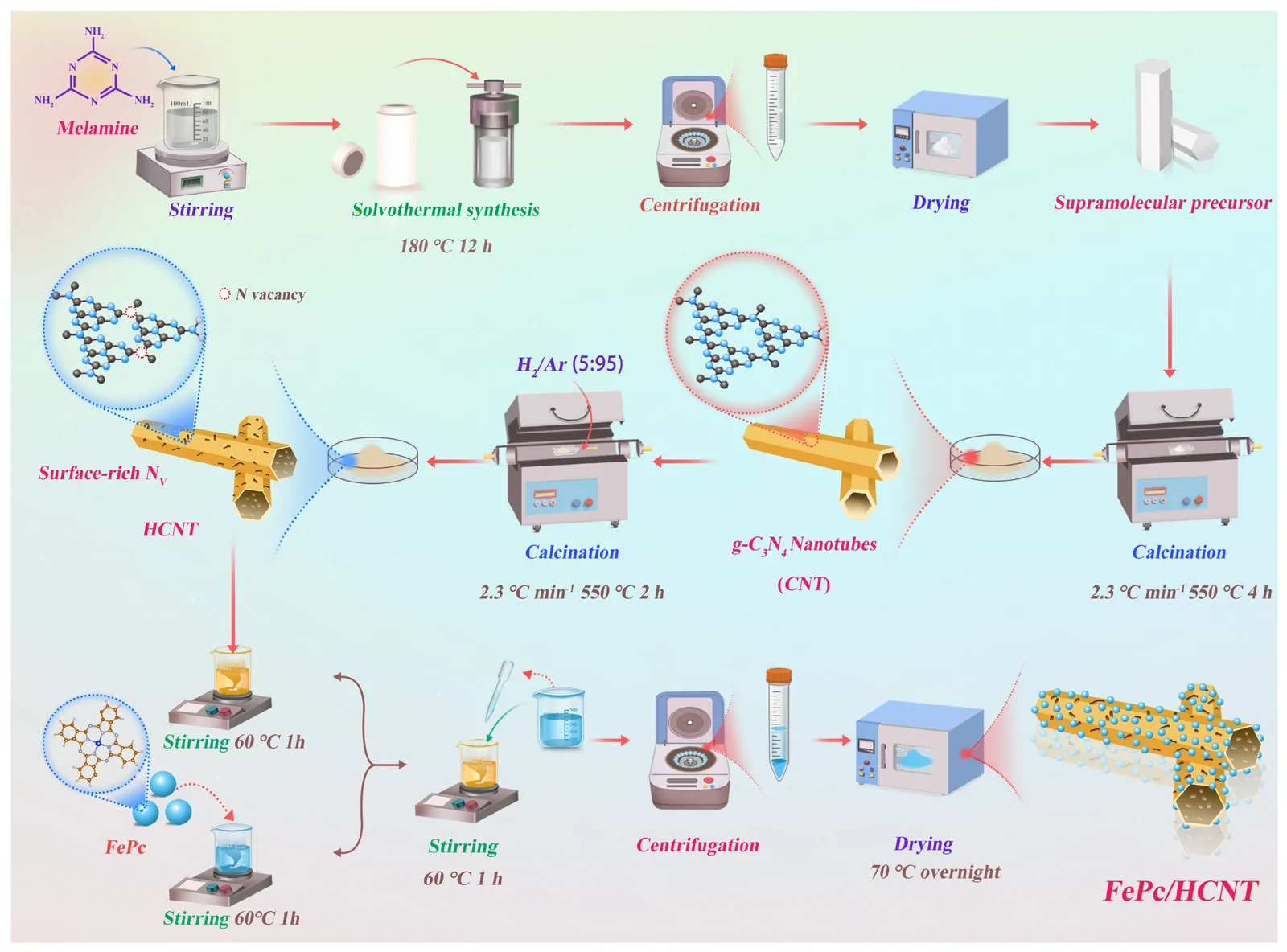
Schematic illustration of the synthetic procedure for FePc/HCNT.

As illustrated in Scheme 1, the tubular g-C_3_N_4_ (CNT) was first synthesized using an optimized method based on previous reports [25]. Specifically, melamine was self-assembled into rod-like precursors under hydrothermal conditions, which were subsequently calcined under an Ar atmosphere to obtain CNT. The as-prepared CNT was then subjected to a secondary calcination under a H_2_/Ar reducing atmosphere to yield HCNT with rich nitrogen vacancies on its surface. Finally, the FePc/HCNT composite was obtained by integrating FePc with the as-obtained HCNT. The digital optical images of CNT, HCNT, and FePc/HCNT-X are shown in Figure S2.

**Figure 1:**
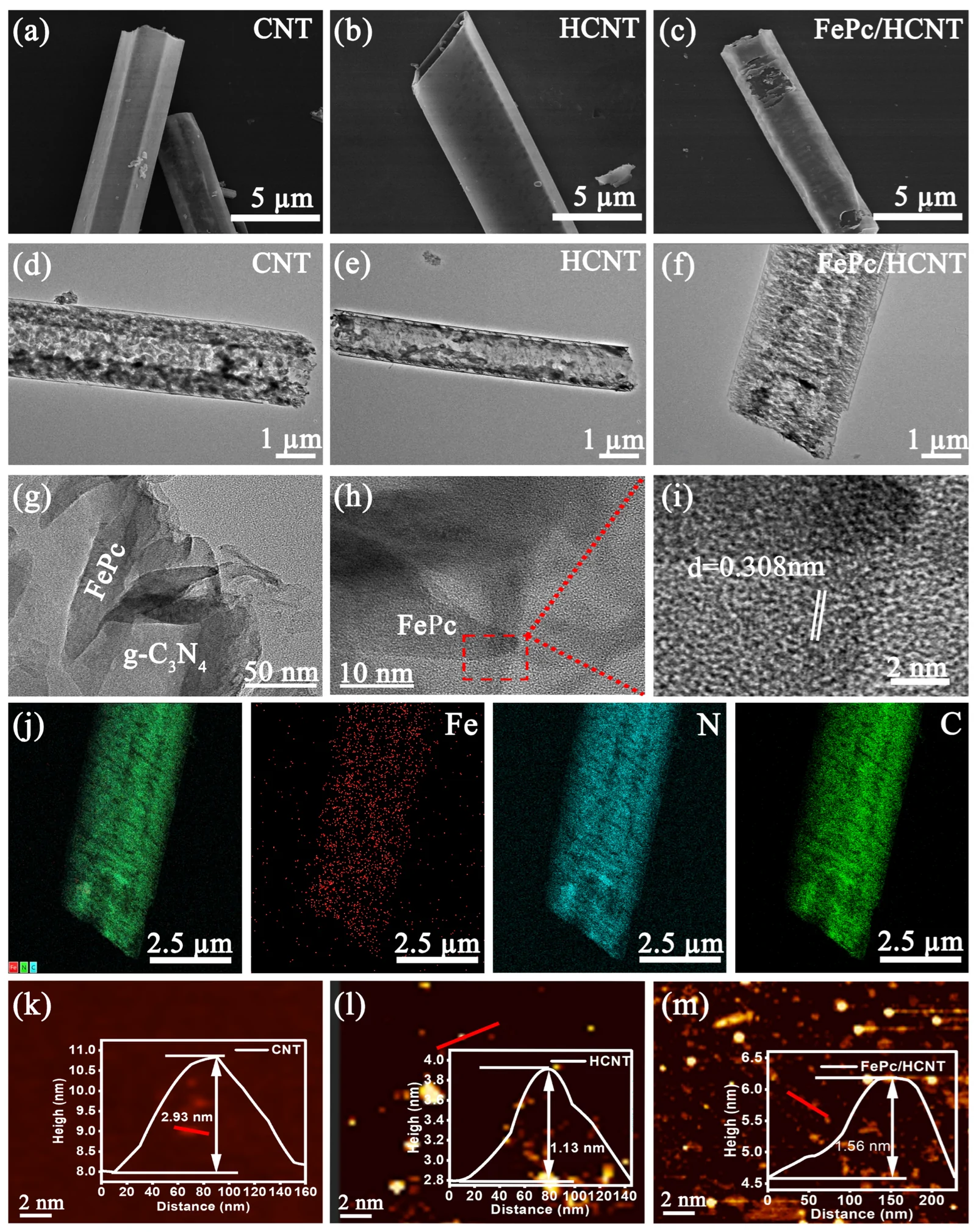
The SEM images of (**a**) CNT, (**b**) HCNT, (**c**) FePc/HCNT; The TEM images of (**d**) CNT, (**e**) HCNT, (**f**) FePc/HCNT; (**g**–**i**) HRTEM and (**j**) elements mapping of FePc/HCNT; AFM images of (**k**) CNT, (**l**) HCNT, (**m**) FePc/HCNT.

The surface morphologies of the samples were examined by scanning electron microscopy (SEM). As shown in Figure S3, the tubular g-C_3_N_4_ precursor exhibits a regular rod-like structure. In Figure 1a, CNT displays a well-defined hollow tubular structure. After H_2_/Ar heat treatment, HCNT retains the tubular morphology (Figure 1b). Upon the introduction of FePc, no significant morphological change is observed for FePc/HCNT, indicating the successful formation of a tubular composite (Figure 1c). Transmission electron microscopy (TEM) images (Figure 1d–f) further confirm the tubular structures of CNT, HCNT, and FePc/HCNT. High-resolution TEM (HRTEM) images (Figure 1g–i) reveal that FePc forms willow leaf-like nanostructures covering the tubular g-C_3_N_4_ [26]. Meanwhile, clear lattice fringes with an interplanar spacing of 0.308 nm are observed in the TEM images of FePc/HCNT, confirming the presence of FePc. Elemental mapping (Figure 1j) shows the uniform distribution of C, N, and Fe elements in the FePc/HCNT catalyst, further demonstrating the highly dispersed loading of FePc on the HCNT surface. Notably, atomic force microscopy (AFM) images (Figure 1k,l) reveal that HCNT exhibits a thinner tubular structure (1.13 nm), which is significantly thinner than that of CNT (2.93 nm), suggesting that the secondary calcination treatment under a H_2_/Ar atmosphere plays a “peeling” role in thinning the tube walls. Furthermore, the AFM image of FePc/HCNT (Figure 1m) shows a thickness of approximately 1.56 nm, which is slightly thicker than that of HCNT. This increase is likely attributable to the layer-by-layer stacking of FePc molecules on the HCNT surface. The SEM images of bulk g-C_3_N_4_ (CN) and sheet-like g-C_3_N_4_ (CNS) are shown in Figure S4. The dense morphology, along with the stacked sheet-like structure, is not favorable for mass transfer or sufficient contact with pollutant molecules in the catalytic system.

**Figure 2:**
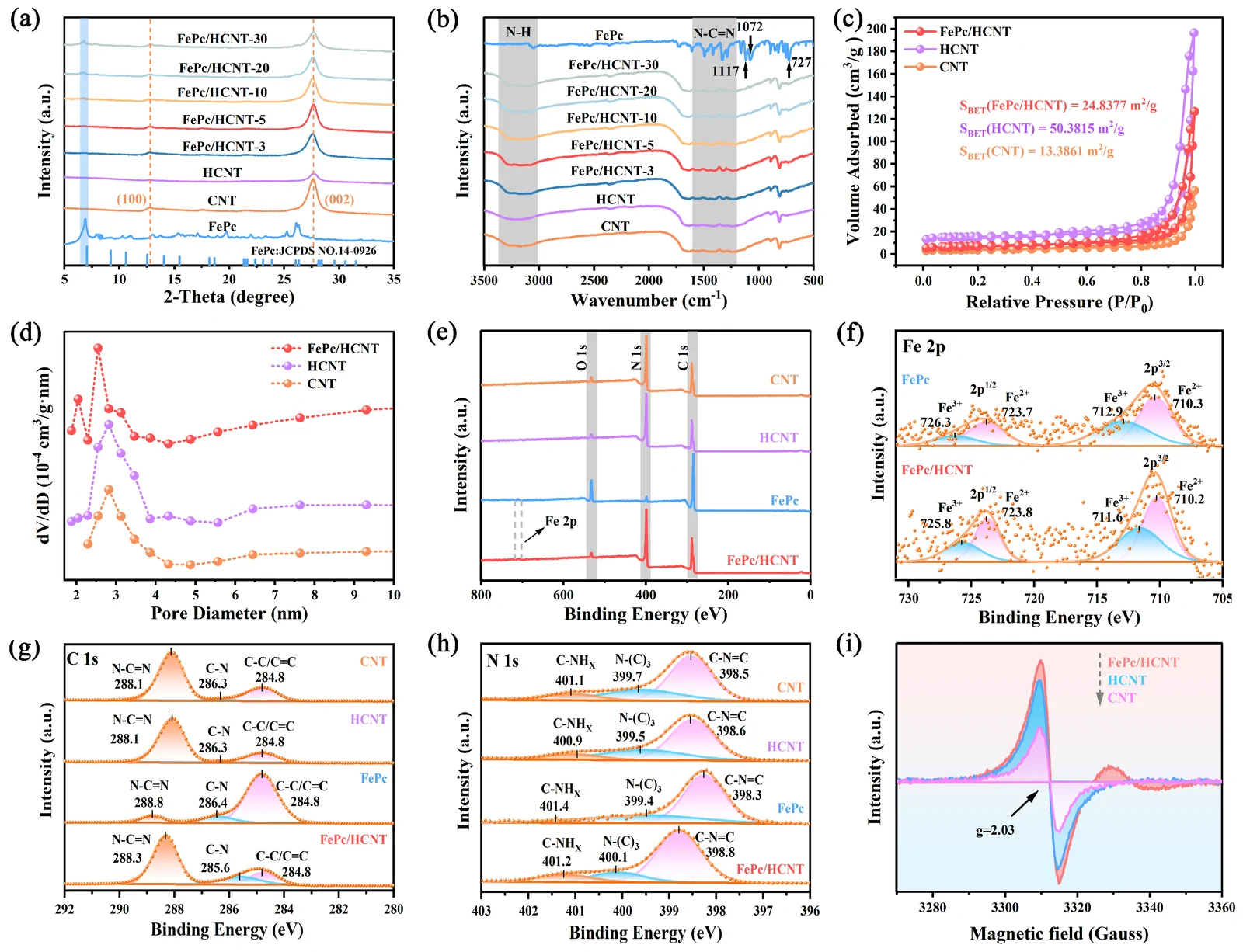
(**a**) XRD spectra, (**b**) FTIR spectra, (**c**) N_2_ adsorption–desorption isotherms, (**d**) pore size distributions, (**e**) XPS survey spectra, (**f**) high-resolution spectra of Fe 2p, (**g**) C 1s, (**h**) N 1s, (**i**) EPR of FePc, CNT, HCNT and FePc/HCNT-X samples.

The crystal structures of the as-prepared catalysts were characterized by X-ray diffraction (XRD). As shown in Figure 2a, the XRD patterns of CNT and HCNT exhibit two distinct diffraction peaks, which correspond to the (100) and (002) lattice planes of g-C_3_N_4_, respectively [27]. The diffraction peak observed at 6.8° is attributed to the characteristic signal of the (200) plane of the single α-phase of FePc [28]. Notably, in the XRD patterns of the composite photocatalysts with varying FePc loadings (FePc/HCNT-3, FePc/HCNT-5, FePc/HCNT-10, FePc/HCNT-20, and FePc/HCNT-30), the characteristic diffraction peaks of g-C_3_N_4_ are clearly present. Moreover, with increasing FePc loading, the diffraction peaks corresponding to FePc gradually intensify. These observations collectively indicate that FePc has been successfully and controllably integrated with HCNT.

The Fourier transform infrared (FTIR) spectra of the as-prepared samples are presented in Figure 2b. For CNT and HCNT, the broad peak in the region of 3042–3246 cm^−1^ corresponds to the stretching vibration of N-H bonds, while the strong vibrational signal observed at around 808 cm^−1^ is attributed to the bending vibration of the triazine rings in g-C_3_N_4_. Additionally, the peaks located in the range of 1200–1600 cm^−1^ are assigned to the typical stretching vibrations of the heterocyclic moiety of g-C_3_N_4_ [29]. For the pristine FePc, the peaks between 1235 and 1635 cm^−1^ are associated with its skeletal stretching and molecular deformation [23,30]. Meanwhile, characteristic vibrational peaks of the FePc molecular structure are also observed at 727, 1072, and 1117 cm^−1^. Notably, all major characteristic bands of g-C_3_N_4_ are well preserved in the spectra of the FePc/HCNT composites. Moreover, the characteristic peaks of FePc can be clearly identified in the composites and gradually intensify with increasing FePc loading, although the peaks in the range of 1235–1635 cm^−1^ are difficult to distinguish due to overlap with those of g-C_3_N_4_. Collectively, these results confirm the successful integration of FePc with HCNT.

The Brunauer–Emmett–Teller (BET) surface areas and pore size distributions of CNT, HCNT, and FePc/HCNT are presented in Figure 2c,d, and the detailed specific surface area values are summarized in Table S1. Among all the samples, HCNT exhibits the highest specific surface area (50.3815 m^2^/g), which is approximately 4 times that of CNT. Notably, the FePc/HCNT composite shows a slightly lower specific surface area than HCNT, which initially decreases and then increases with increasing FePc loading. This trend can be reasonably explained as follows: at lower loadings, FePc molecules adhere to the HCNT surface and partially block the pore structure, leading to a reduction in surface area. In contrast, excessive FePc loading results in disordered stacking among FePc molecules, which introduces additional void spaces and consequently contributes to an increased specific surface area [31,32]. This hypothesis is further supported by the pore size distribution results shown in Figure 2d. After hydrogen heat treatment, CNT develops more pores in the 2–4 nm range. Following the attachment of FePc molecules onto HCNT, the number of 3–4 nm pores decreases, whereas pores larger than 5 nm become more abundant. This retained large specific surface area and porous structure of FePc/HCNT are beneficial for promoting the adsorption and activation of reactant molecules.

To investigate the elemental composition and chemical states of the prepared samples, X-ray photoelectron spectroscopy (XPS) was employed. As depicted in Figure 2e, the distinct signals corresponding to C, N, O, and Fe elements are observed, confirming the successful incorporation of FePc [33]. Further insight into the chemical environment of Fe is provided by the high-resolution Fe 2p spectra, which can be deconvoluted into four peaks (Figure 2f). Specifically, the peaks at 710.3 eV and 723.7 eV are assigned to Fe^2+^ 2p^3/2^ and Fe^2+^ 2p^1/2^, respectively, while those at 712.9 eV and 726.3 eV correspond to Fe^3+^ 2p^3/2^ and Fe^3+^ 2p^1/2^ [34]. These assignments reveal the coexistence of Fe^3+^ even in pristine FePc, which is attributable to the electron-withdrawing nature of the phthalocyanine ring [35]. Nonetheless, Fe^2+^ remains the predominant species, accounting for 57% of the total Fe content based on the peak area ratio. Upon loading FePc onto HCNT, the Fe^2+^ proportion slightly decreases to 53% in the FePc/HCNT composite. This variation points to the existence of strong π-π interactions between FePc and the HCNT support. More importantly, following the integration of FePc into HCNT, the Fe 2p binding energies exhibit a subtle negative shift, implying interfacial coordination and electron transfer from the Fe centers to the HCNT substrate [36].

Figure 2g presents the C 1s XPS spectra of CNT, HCNT, FePc, and FePc/HCNT, all of which exhibit three main peaks. The peak at 284.8 eV is assigned to graphitic carbon (C-C/C=C), while the second peak located at 286.3 eV corresponds to sp^3^-hybridized carbon bonds (C-N). The third peak at 288.1 eV is attributed to sp^2^ carbon atoms bonded to nitrogen within the triazine units (N-C=N) [37]. Notably, compared to CNT, HCNT, and bare FePc, the FePc/HCNT composite shows a markedly higher intensity of the C-N peak, implying significant charge transfer and rearrangement of electron density between the π-conjugated systems of FePc and HCNT. This observation suggests the formation of strong interfacial interactions rather than weak physical electrostatic adsorption, confirming the successful anchoring of FePc onto the HCNT surface [29].

As shown in the N 1s spectra (Figure 2h), three deconvoluted peaks are observed at 398.6, 399.7, and 401.1 eV [38], corresponding to sp^2^-hybridized nitrogen (C-N=C), tertiary nitrogen in N-(C)_3_, and C-N-H groups, respectively. It is worth noting that the N-(C)_3_ peak in HCNT shifts slightly toward a lower binding energy (399.5 eV), suggesting the possible presence of rich nitrogen vacancies (N_v_s), which may influence the local π-electron arrangement around the vacancy sites [39]. More importantly, upon the integration of FePc, all three N 1s peaks in FePc/HCNT shift slightly toward higher binding energies relative to those of HCNT, among which the C-N=C and N-(C)_3_ peaks exhibit the most pronounced shifts. This observation indicates an increase in electron density around the nitrogen atoms. Taken together with the negative shifts of the Fe 2p and C 1s peaks in FePc, as well as the positive shifts of the C-N=C and N-(C)_3_ peaks in HCNT, it can be clearly inferred that strong π-π conjugation and electronic coupling exist between the FePc macrocycle and the HCNT framework. Such interactions facilitate interfacial charge transfer and stabilize the FePc molecules on the support. Consequently, these π-π stacking interactions provide abundant anchoring sites for FePc and effectively suppress its self-aggregation, thereby enabling a highly molecular-level dispersion of FePc [40].

To verify the presence of abundant nitrogen vacancies (N_v_s) on the HCNT surface, electron paramagnetic resonance (EPR) spectroscopy was employed to evaluate the vacancy concentration in the as-prepared samples. As shown in Figure 2i, CNT, HCNT, and FePc/HCNT all exhibit a distinct EPR signal with a g-value of 2.03. Notably, the significantly stronger EPR intensity observed for HCNT compared to CNT indicates that a greater number of vacancies are generated following the hydrogen heat treatment [32]. Furthermore, the EPR signal of FePc/HCNT is even stronger than that of HCNT. This enhancement can be attributed to the presence of localized unpaired electrons from the Fe and N species in the FePc molecules, as well as the electronic coupling between FePc and the HCNT support.

### 3.2. Photocatalytic Performance

**Figure 3:**
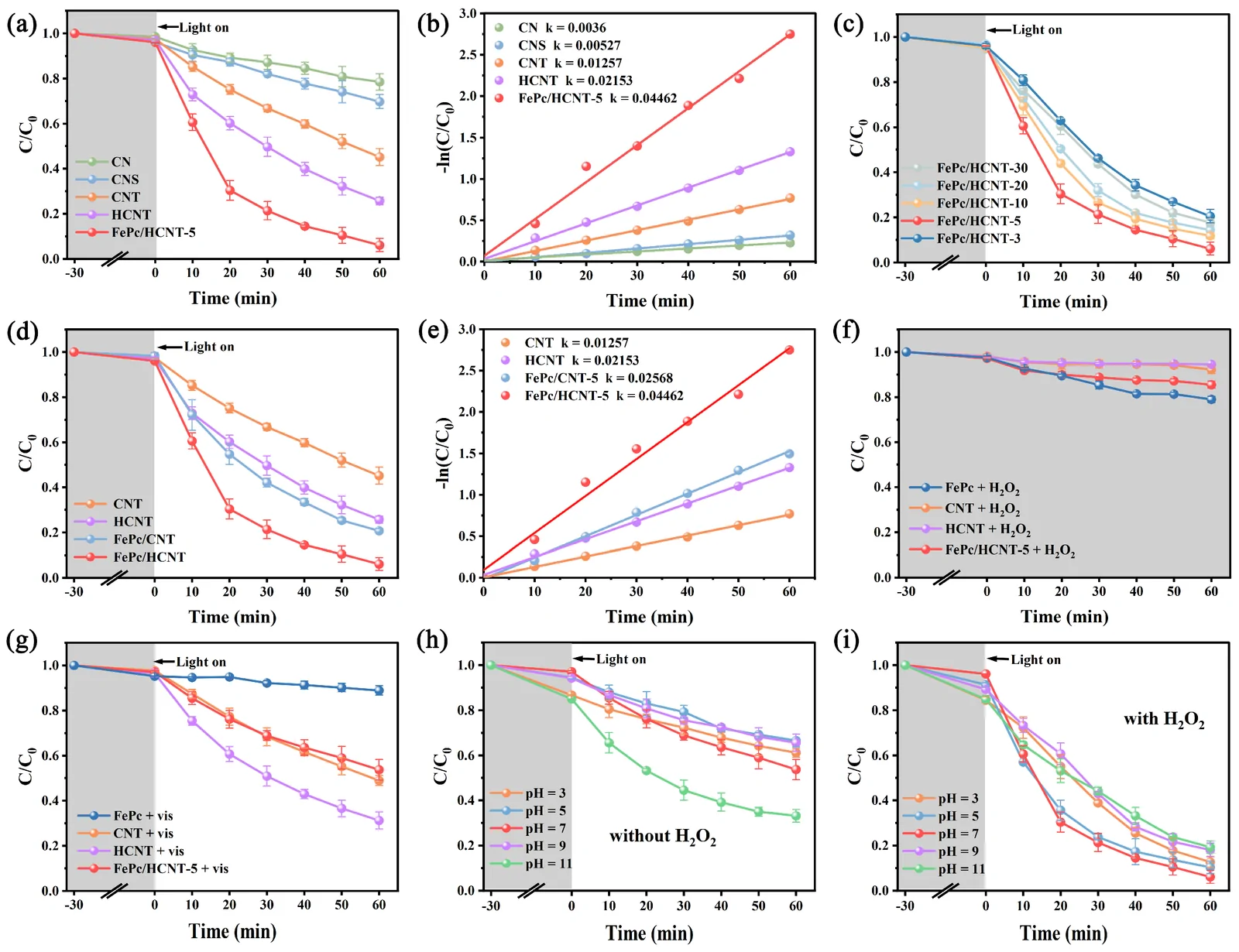
(**a**) Degradation efficiency and (**b**) corresponding kinetic curves of OTC over CN, CNS, CNT, HCNT, and FePc/HCNT-5 samples (OTC = 40 mg·L^−1^, catalyst = 0.02 g, pH = 7, H_2_O_2_ = 15 mmol/L). (**c**) Effect of FePc loading on the degradation efficiency of FePc/HCNT composites (OTC = 40 mg·L^−1^, catalyst = 0.02 g, pH = 7, H_2_O_2_ = 15 mmol/L). (**d**,**e**) Photodegradation efficiency curves and kinetic of CNT, HCNT, and their corresponding FePc-loaded composite samples (OTC = 40 mg·L^−1^, catalyst = 0.02 g, pH = 7, H_2_O_2_ = 15 mmol/L). (**f**) OTC degradation under only H_2_O_2_ and (**g**) only light irradiation over different samples. (**h**,**i**) Degradation efficiency curves under different pH conditions in the presence or absence of H_2_O_2_ (OTC = 40 mg·L^−1^, catalyst = 0.02 g, H_2_O_2_ = 15 mmol/L).

To systematically evaluate the catalytic activity of the as-prepared samples, their efficiency in activating H_2_O_2_ for oxytetracycline (OTC) degradation was investigated under visible light irradiation. As shown in Figure S5, FePc, CNT, HCNT, and FePc/HCNT all exhibited weak adsorption toward OTC and reached adsorption equilibrium within 30 min, with adsorption efficiencies below 5% and only small differences among the samples. Therefore, physical adsorption makes only a minor contribution to the decrease in OTC concentration, and the observed OTC removal under light irradiation mainly originates from the photo-Fenton degradation process. As presented in Figure 3a, the degradation performance of pristine bulk g-C_3_N_4_ (CN) and g-C_3_N_4_ nanosheets (CNS) is significantly lower than that of CNT, indicating that the tubular morphology substantially enhances the catalytic activity of g-C_3_N_4_. Moreover, HCNT, which possesses rich N_v_s on its surface, exhibits further improved OTC degradation performance, increasing from 54.7% to 74.3%. This result highlights the critical role of surface nitrogen vacancies in boosting the catalytic activity of g-C_3_N_4_. Notably, the FePc/HCNT-5 composite, obtained by anchoring FePc molecules onto HCNT surface, achieves a high OTC degradation efficiency of 94% within 60 min, fully demonstrating the effectiveness of the modification strategy of “Tubular structure engineering–Nitrogen vacancy introduction–FePc incorporation”. As presented in Figure 3b, the degradation kinetic constants of CN, CNS, CNT, HCNT, and FePc/HCNT are determined to be 0.0036, 0.00527, 0.01257, 0.02153, and 0.04462 min^−1^, respectively. Among them, FePc/HCNT exhibits the highest kinetic constant, which is approximately 11 times that of CN, consistent with the results shown in Figure 3b. To examine whether the enhanced activity of FePc/HCNT arises from a simple additive contribution of FePc and HCNT, a FePc + HCNT physical mixture with the same FePc content as FePc/HCNT-5 was used as a control. As shown in Figure S6, the physical mixture exhibited much lower OTC degradation efficiency than FePc/HCNT-5 under identical photo-Fenton conditions, confirming the important role of the FePc/HCNT interfacial interaction in the enhanced catalytic activity. To optimize the key parameters of the catalytic reaction, the effects of FePc loading amount, initial pollutant concentration, catalyst and H_2_O_2_ dosage, and initial solution pH were systematically investigated. As shown in Figure 3c, the loading amount of FePc on the HCNT surface significantly influences the catalytic performance of the FePc/HCNT composites. Among all the tested samples, FePc/HCNT-5 exhibits the optimal catalytic activity, benefiting from an appropriate FePc loading amount. Both insufficient and excessive FePc loadings are detrimental to the catalytic performance. A low loading provides too few active sites, while a high loading induces aggregation of FePc molecules on the HCNT surface, which blocks tubular channels, impedes mass transfer, reduces light transmittance, and shields active sites from one another.

To further verify the significant contribution of surface-rich nitrogen vacancies to the catalytic activity, a control sample (FePc/CNT-5) was prepared by loading FePc onto CNT using the same method as for FePc/HCNT-5. The OTC degradation performance of FePc/CNT-5 is compared with that of FePc/HCNT-5 in Figure 3d,e. Evidently, simply loading FePc onto CNT results in only a limited improvement in catalytic activity. The reaction rate constant of FePc/HCNT-5 (0.04462 min^−1^) is nearly twice that of FePc/CNT-5 (0.02568 min^−1^). These results underscore the necessity of introducing abundant surface nitrogen vacancies through secondary calcination of CNT under thea H_2_/Ar atmosphere, and more importantly, highlight the synergistic effect between surface nitrogen vacancies and FePc incorporation. To further verify the existence of a photocatalysis-Fenton synergistic effect, the OTC removal efficiency of FePc/HCNT and other reference samples was evaluated under Fenton-only and photocatalysis-only conditions, with the results shown in Figure 3f,g. In the Fenton-only system (with H_2_O_2_ but without light, Figure 3f), only a small amount of OTC was degraded for all samples, indicating that these materials have low intrinsic activity for H_2_O_2_ activation and the light irradiation is essential for initiating an efficient catalytic reaction. In the photocatalysis-only system (with light but without H_2_O_2_, Figure 3g), HCNT exhibits the best OTC removal efficiency (approximately 70%), outperforming FePc/HCNT, CNT, and pristine FePc. This result indicates the key role of abundant nitrogen vacancies (N_v_s) on the HCNT surface in enhancing its photocatalytic performance. Obviously, the catalytic activities of both the Fenton-only and photocatalysis-only systems are lower than that of the FePc/HCNT + H_2_O_2_ + Vis system, which achieves an OTC removal efficiency of 94%. These findings demonstrate the critical contributions of surface nitrogen vacancies and heterostructure (FePc/HCNT) construction to the efficient photocatalysis-Fenton mechanism of the FePc/HCNT composite catalyst.

The effect of the initial pH of the OTC solution on the catalytic efficiency was studied. As shown in Figure 3h, in the photocatalytic system (without H_2_O_2_), FePc/HCNT-5 shows the best degradation performance at pH = 11, where its ability to adsorb OTC molecules is also better than under neutral or acidic conditions. This is because that increase in pH solution alters the surface charge polarity of the catalyst, thereby changing its adsorption affinity toward OTC molecules and ultimately impacting the photocatalytic performance. In contrast, FePc/HCNT-5 gives the best degradation performance under neutral conditions (pH = 7) in the photo-Fenton system (with H_2_O_2_), reaching an OTC degradation efficiency as high as 94% within 60 min (Figure 3i). A slight drop in degradation efficiency is observed under alkaline conditions. This decrease is attributed to the poor stability and short lifetime of ∙OH radicals in alkaline environments [41,42]. These results (Figure 3h,i) suggest that the active species and reaction mechanisms in the photo-Fenton system are quite different from those in the photocatalytic system.

**Figure 4:**
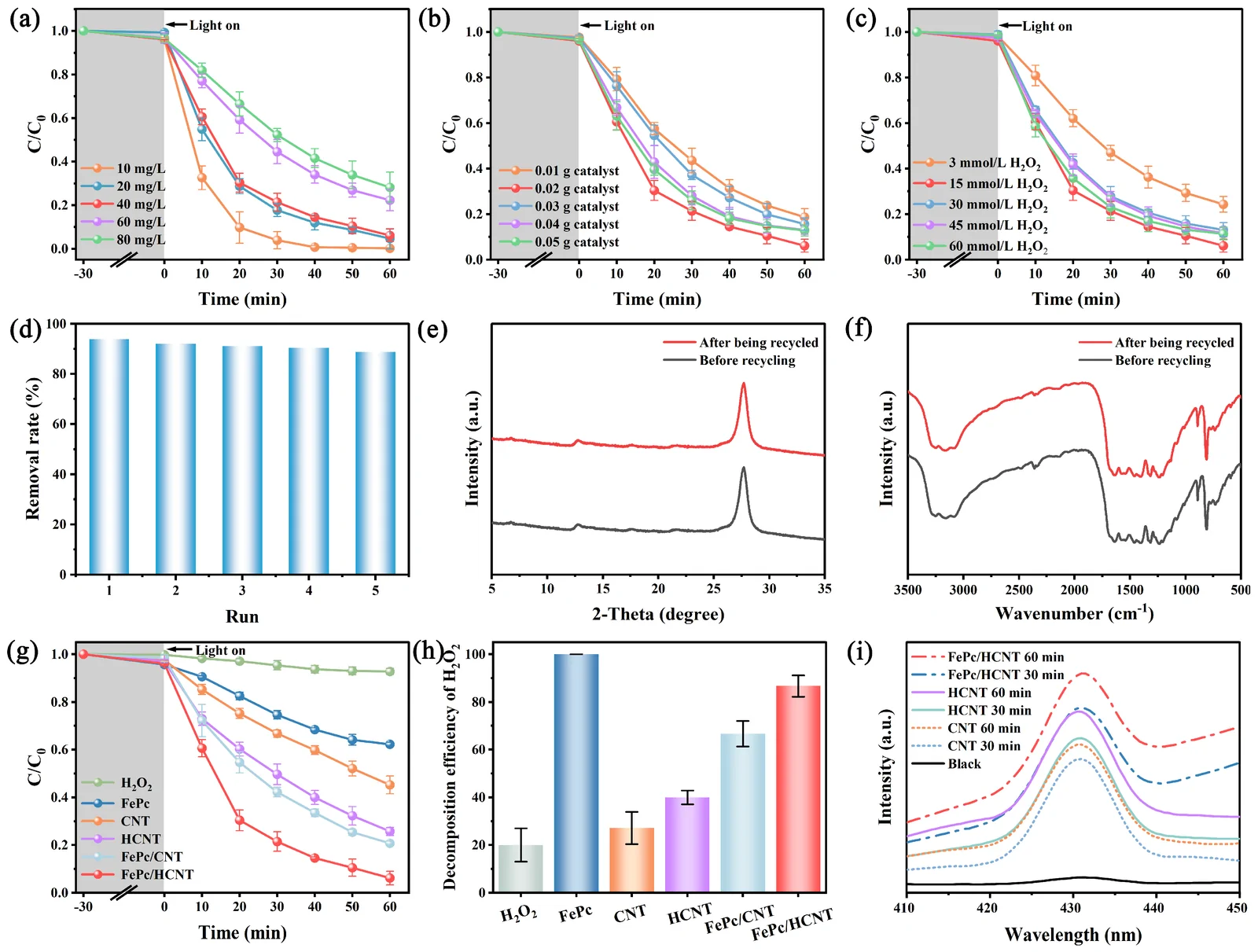
Effects of (**a**) initial OTC concentration, (**b**) catalyst dosage, and (**c**) H_2_O_2_ dosage on the OTC removal efficiency of FePc/HCNT-5 in the photo-Fenton system. (**d**) Cyclic degradation efficiency of FePc/HCNT-5 for OTC under visible light (OTC = 40 mg·L^−1^, catalyst = 0.02 g, pH = 7, H_2_O_2_ = 15 mmol/L). (**e**) XRD patterns and (**f**) FT-IR spectra of fresh and used FePc/HCNT-5. (**g**) Component-dependent OTC degradation performance of FePc, CNT, HCNT, and FePc/HCNT in the photo-Fenton system, and (**h**) corresponding H_2_O_2_ consumption efficiency. (**i**) Comparison of •OH radical generation capability among CNT, HCNT, FePc/HCNT, and blank systems under visible-light irradiation for 30 and 60 min.

The influence of the initial pollutant concentration on the OTC degradation performance is presented in Figure 4a. Although the degradation efficiency gradually declines with increasing initial concentration, a notable removal rate of approximately 70% is still achieved even at a high concentration of 80 mg/L. This result suggests that FePc/HCNT-5 holds promise for the treatment of high-concentration antibiotic wastewater. Meanwhile, the influence of catalyst dosage on the OTC degradation efficiency is presented in Figure 4b. The best OTC removal was achieved at a dosage of 0.02 g. Further increasing the catalyst dosage may weaken the system’s ability to absorb and utilize visible light. As a result, the degradation performance gradually declines. Figure 4c examines the effect of H_2_O_2_ dosage on the OTC removal efficiency. The results show that the removal efficiency first increases and then decreases with increasing H_2_O_2_ dosage. An appropriate increase in H_2_O_2_ dosage generates more reactive oxygen species (ROS), thereby accelerating the oxidative degradation of the pollutant. However, an excessive amount of ROS is unfavorable for the reaction, as a high concentration of H_2_O_2_ can inhibit the reduction of Fe^3+^ to Fe^2+^ [43]. The reusability of FePc/HCNT-5 was evaluated through consecutive degradation cycles (Figure 4d). The results show that the OTC removal efficiency remained above 80% after five cycles. Furthermore, the XRD patterns and FT-IR spectra of the used FePc/HCNT-5 were nearly identical to those of the fresh sample (Figure 4e,f), further confirming its structural stability of FePc/HCNT-5 [44].

Furthermore, the Fe leaching behavior of FePc/HCNT-5 was investigated in the presence and absence of H_2_O_2_ during the photo-Fenton reaction (Figure S7). In the absence of H_2_O_2_, the Fe leaching concentration was only 0.105 mg L^−1^, corresponding to an Fe leaching rate of approximately 2.19%. In contrast, the addition of H_2_O_2_ increased the Fe leaching concentration to 0.285 mg L^−1^, corresponding to an Fe leaching rate of approximately 5.95%. The slight increase in Fe dissolution after H_2_O_2_ addition can be attributed to the involvement of Fe^2+^/Fe^3+^ redox cycling during the photo-Fenton process. It is generally accepted that the reversible oxidation and reduction of Fe species are essential for Fenton reactions; however, the continuous valence-state transformation may inevitably induce partial structural instability of Fe-based active sites, resulting in increased Fe leaching [45].

To investigate the regulation of surface N vacancies in HCNT and their influence on the photo-Fenton degradation performance toward OTC, CNT was thermally treated under an H_2_/Ar atmosphere for 1, 2, and 4 h to obtain HCNT samples with different N-vacancy concentrations, denoted as HCNT-1h, HCNT-2h, and HCNT-4h, respectively. As shown in Figure S8, all three samples exhibited a distinct EPR signal at a g value of approximately 2.03, and the signal intensity gradually increased with prolonged thermal treatment time. This result indicates that the concentration of surface N vacancies in HCNT increased progressively with increasing treatment duration under the H_2_/Ar atmosphere. The OTC degradation performances of the as-prepared HCNT samples are presented in Figure S9. The catalytic activity exhibited a volcano-type dependence on the N-vacancy concentration, with HCNT-2h achieving the highest OTC removal efficiency, whereas HCNT-1h and HCNT-4h exhibited comparable but inferior activities. After further coupling with FePc, the corresponding FePc/HCNT catalysts displayed an identical activity sequence (FePc/HCNT-2h > FePc/HCNT-4h > FePc/HCNT-1h), demonstrating that the optimized N-vacancy concentration plays a critical role in regulating the photo-Fenton catalytic performance. These results suggest that an appropriate density of surface N vacancies is favorable for enhancing catalytic activity, whereas excessive defect generation may lead to performance deterioration. The decreased activity of HCNT-4h and FePc/HCNT-4h could be related to the over-abundance of structural defects, which may induce unfavorable electronic perturbation or compromise the structural integrity of the carbon framework, thereby impairing the catalytic process.

### 3.3. Identification of Active Sites and Synergistic Effects

To elucidate the specific contributions of individual components and clarify the synergistic effects within the FePc/HCNT heterojunction, a series of comparative experiments were systematically performed (Figure 4g,h). To evaluate the hydroxyl radical (•OH) generation capability during the photo-Fenton process, terephthalic acid (TA) was employed as a fluorescent probe, and the time-dependent formation of •OH radicals was monitored by photoluminescence (PL) spectroscopy (Figure 4i).

As shown in Figure 4g, negligible OTC degradation was observed in the presence of H_2_O_2_ alone under light irradiation, revealing the limited oxidative capability of H_2_O_2_ without catalyst assistance. In contrast, FePc exhibited effective photo-assisted H_2_O_2_ activation capability (Figure 4h), resulting in moderate OTC degradation efficiency (<40%) under photo-Fenton conditions. The considerable amount of H_2_O_2_ confirms that FePc serves as the primary catalytic center for H_2_O_2_ activation.

Compared with FePc, pristine CNT displayed limited H_2_O_2_ activation capability (Figure 4h), and its OTC degradation activity mainly originated from its intrinsic photocatalytic oxidation process rather than H_2_O_2_-mediated oxidation (Figure 3g). The relatively weak •OH generation ability of CNT (Figure 4i) further demonstrated its limited contribution to oxidative species formation. After introducing abundant surface nitrogen vacancies, HCNT exhibited enhanced H_2_O_2_ activation capability compared with CNT (Figure 4h), accompanied by increased •OH generation (Figure 4i). This improvement is likely associated with the modified surface electronic structure and chemical environment induced by N vacancies, which may provide favorable conditions for H_2_O_2_ activation. However, the OTC degradation performance of HCNT remained mainly dependent on its intrinsic photocatalytic oxidation pathway rather than H_2_O_2_-mediated oxidation (Figure 3g).

Upon constructing the FePc/CNT heterojunction, the intimate interfacial coupling between FePc active centers and CNT significantly enhanced H_2_O_2_ activation efficiency. More importantly, replacing CNT with N-vacancy-rich HCNT further optimized the interfacial electronic structure, enabling FePc/HCNT to achieve the highest H_2_O_2_ activation capability and •OH generation efficiency among all investigated catalysts.

These comparative results indicate that FePc provides the major contribution to H_2_O_2_ activation, while HCNT primarily functions as a defect-engineered tubular photocatalytic platform for the additional generation and separation of photogenerated carriers. The introduction of nitrogen vacancies also enhances the H_2_O_2_ activation capability of HCNT relative to CNT, suggesting that the carbon nitride component may additionally participate in this process. Upon coupling FePc with HCNT, the interfacial interaction promotes both H_2_O_2_ activation and photocatalytic redox reactions, thereby enhancing the overall photo-Fenton activity.

### 3.4. Photoelectrochemical Properties

**Figure 5:**
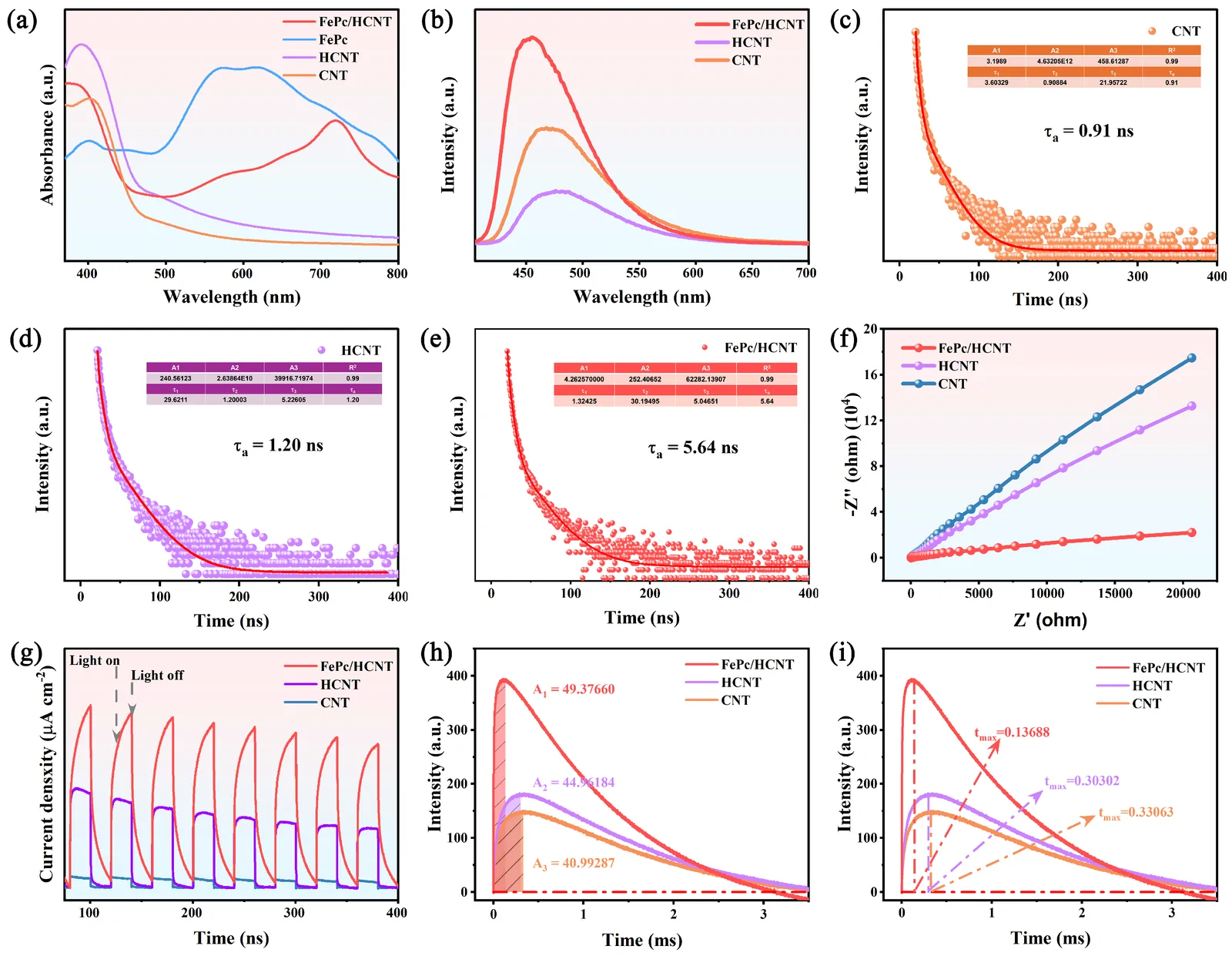
(**a**) UV–vis DRS, (**b**) PL spectra, (**c**–**e**) TRPL spectra of CNT, HCNT, and FePc/HCNT; (**f**) EIS, (**g**) photocurrent response, (**h**) extracted charge amount (A), and (**i**) maximum charge extraction time (t_max_) from TPV for CNT, HCNT, and FePc/HCNT.

The light absorption properties of the as-prepared samples were investigated using ultraviolet–visible diffuse reflection spectroscopy (DRS), and the results are presented in Figure 5a. Compared with bulk g-C_3_N_4_ (CN), the tubular CNT exhibits better UV–vis light utilization efficiency (Figure S10), justifying the selection of a hollow tubular morphology as a favorable modification strategy for g-C_3_N_4_. Moreover, HCNT shows increased light absorption intensity in the range of 400–800 nm relative to CNT. This enhancement is likely attributable to the thinner tube walls, larger specific surface area, and broader pore size distribution of HCNT resulting from the secondary calcination treatment (Figure 2c,d). As a photosensitizer, pristine FePc displays intrinsically strong absorption in the UV–vis region, which originates from the π-π* transitions between the high and low-energy levels of its conjugated electronic structure [46]. Therefore, from the perspective of improving the light utilization efficiency of the composite material, it is reasonable to combine FePc as a photosensitizer with HCNT to extend the spectral response range. Based on the UV–Vis DRS data, the band gaps (Eg) of CNT, HCNT, and FePc were calculated using the Tauc-plot method (Equation (3)). The results are shown in Figure S11a–c, indicating that the band gaps of CNT, HCNT, and FePc are 2.68 eV, 2.75 eV, and 2.03 eV, respectively. Notably, the introduction of surface nitrogen vacancies (N_v_s) slightly alters the local electron distribution environment on the HCNT surface, resulting in a subtle tuning of its band gap [47].

(3) $${\left(\alpha h\upsilon \right)}^{1/n}=A\left(h\upsilon -E_{g}\right)$$

The valence band (VB) positions of the catalysts were determined by XPS valence band spectra combined with Equation (4), and the results are shown in Figure S12. The valence band edge (E_VB_) values of CNT, HCNT, and FePc are 2.21, 1.94, and 0.86 V, respectively. Here, ϕ represents the work function of the instrument (4.39 eV), and 4.44 eV corresponds to the vacuum level [48]. Using the equation $E_{\mathrm{CB}}\;=\;E_{\mathrm{VB}}\;-\;E_{g}$, the conduction band (CB) positions of CNT, HCNT, and FePc were calculated to be −0.47, −0.81, and −1.17 V, respectively. Based on these results, the band energy diagrams of CNT, HCNT, and FePc are illustrated in Figure S13.

(4) $$\mathrm{VB}\left(\mathrm{vs}\cdot \;\mathrm{NHE}\right)=\phi +{\mathrm{VB}}_{\mathrm{XPS}}-4.44\;\mathrm{eV}\;$$

The separation efficiency of photogenerated charge carriers was assessed by PL spectroscopy (Figure 5b, excitation at 375 nm). A strong PL signal typically reflects a high recombination rate of electron–hole pairs, limiting electron availability for catalysis. HCNT exhibits a much lower intensity, likely due to the reduced conjugation length caused by nitrogen vacancies (N_v_s) and the strong quantum confinement of its ultrathin porous structure. The FePc/HCNT composite displays an increased PL intensity, possibly resulting from interfacial charge recombination between partially separated carriers from different catalysts [49]. The carrier dynamics were further investigated using time-resolved photoluminescence (TRPL) spectroscopy (Figure 4c–e). The average lifetimes of photogenerated charge carriers in CNT, HCNT, and FePc/HCNT were determined by fitting the decay curves using Equation (5). Notably, FePc/HCNT exhibits a much longer average carrier lifetime (5.64 ns) compared to HCNT (1.20 ns) and CNT (0.91 ns), which is directly correlated with its superior photocatalytic performance [50,51].

(5) $$\tau =\frac{A_{1}\times {\tau_{1}}^{2}+A_{2}\times {\tau_{2}}^{2}+A_{3}\times {\tau_{3}}^{2}}{A_{1}\times \tau_{1}+A_{2}\times \tau_{2}+A_{3}\times \tau_{3}}$$

Transient photocurrent and electrochemical impedance spectroscopy (EIS) measurements were further performed to evaluate the improvements in photogenerated charge separation and transfer efficiency of the FePc/HCNT catalyst. The EIS results in Figure 5f show that the arc radius of FePc/HCNT is notably smaller than those of CNT and HCNT, indicating that the combination of FePc with HCNT effectively enhances charge transfer efficiency. Regarding the photocurrent response (Figure 5g), HCNT exhibits a substantially higher photocurrent intensity than CNT, suggesting that the rich surface nitrogen vacancies (N_v_s) facilitate the efficient separation of photogenerated charge carriers. Moreover, FePc/HCNT displays the strongest photocurrent response among all tested samples, demonstrating that the synergy between the built-in electric field at the heterostructure interface and the nitrogen vacancies further improves the photogenerated charge carrier separation efficiency of the composite [52,53].

Transient photovoltage (TPV) measurements were conducted to probe the spatial charge distribution in the photocatalytic materials under illumination. From the TPV decay profiles, key parameters were extracted: the extracted charge amount (A, Figure 5h), the maximum charge extraction time (t_max_, Figure 5i), and the electron decay constant (τ, Figure S14). The charge extraction capability (A), represented by the integrated area under the TPV curve (shaded region), reflects the catalyst’s ability to generate photogenerated electrons, while τ characterizes the charge recombination kinetics. FePc/HCNT exhibits the highest extracted charge amount (A), indicating a substantially enhanced capacity for photogenerated carrier generation. Meanwhile, the observation that A(HCNT) > A(CNT) further demonstrates that nitrogen vacancies (N_v_s) significantly contribute to the improved photogenerated charge capture ability [54]. The t_max_ value of FePc/HCNT is the shortest (0.13688 ms), suggesting a high rate of photogenerated charge generation and extraction [55]. Moreover, FePc/HCNT shows the shortest τ value, which correlates with its fast charge transport, consistent with the EIS results (Figure 5f).

Based on the competition between charge extraction and recombination processes, the effective charge number (n_eff_ was introduced to quantitatively evaluate the overall carrier transport and separation efficiency (Figure S15). This parameter was calculated using Equation (6), with the detailed calculation parameters listed in Table S2. The results show that the composite exhibits the highest n_eff_ value (589.485), which is nearly 2 times greater than that of HCNT (373.074). Therefore, the loading of FePc enables the composite to rapidly collect and transfer a large amount of charge [56].

(6) $$n_{\mathrm{eff}}=\frac{A\times \tau }{t_{\max}}$$

### 3.5. Degradation Mechanisms and Pathways

**Figure 6:**
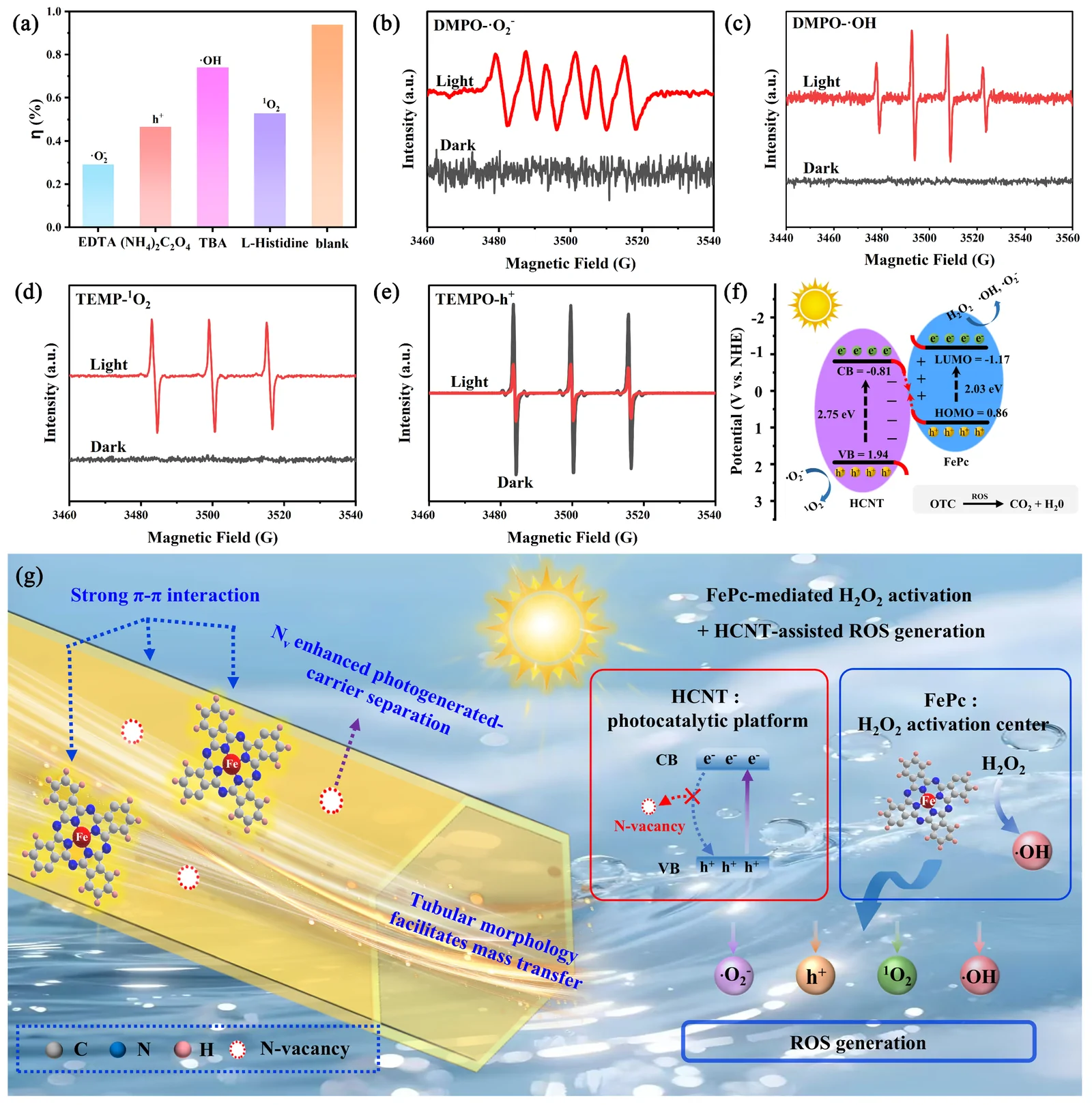
(**a**) Identification of reactive species in the photo-Fenton synergistic system of FePc/HCNT; EPR spectra of (**b**) DMPO-∙O_2_^−^, (**c**) DMPO-∙OH, (**d**) TEMP-^1^O_2_, and (**e**) TEMPO-h^+^ in the photo-Fenton system of FePc/HCNT. (**f**) Schematic illustration of the photo-Fenton mechanism of FePc/HCNT under visible light irradiation. (**g**) Schematic representation of the synergistic roles of FePc and HCNT in enhanced photo-Fenton activity.

To elucidate the oxidation mechanism of OTC degradation over the FePc/HCNT photo-Fenton system, the involved reactive oxygen species (ROS) were first identified by radical scavenging experiments and electron paramagnetic resonance (EPR) measurements. EDTA, (NH_4_)_2_C_2_O_4_, TBA (tert-butyl alcohol), and L-histidine were employed as scavengers to capture ∙O_2_^−^, h^+^, ∙OH, and ^1^O_2_, respectively. As shown in Figure 6a, these reactive species exert varying degrees of influence on the degradation of OTC, indicating that their synergistic action accelerates the OTC degradation rate, with the contribution order following: ∙O_2_^−^ > h^+^ > ^1^O_2_ > ∙OH. EPR measurements were further performed to confirm the generation of these reactive species (Figure 6b–e). No radical signals were detected in dark, whereas clear signals of ∙O_2_^−^, h^+^, ∙OH, and ^1^O_2_ appeared under light irradiation. These results are consistent with the scavenger experiments and demonstrate that OTC degradation proceeds through the cooperative action of multiple light-induced oxidative species rather than through a single dominant oxidation pathway.

The interfacial electronic behavior of FePc/HCNT was further analyzed based on the band structures and previously reported work functions of FePc and g-C_3_N_4_. Previous studies have reported that FePc and g-C_3_N_4_ possess work functions of approximately 4.23 and 4.50 eV, respectively [57,58]. Because of this difference in Fermi levels, charge transfer occurs upon contact between the two materials, i.e., electrons transfer from FePc to g-C_3_N_4_, generating a built-in electric field at the interface and resulting in band bending (Figure 6f). This interpretation is consistent with the XPS results.

Based on the ROS identification results, band alignment analysis, and interfacial electronic interaction, a possible S-scheme-like charge-transfer pathway is proposed for FePc/HCNT (Figure 6f,g). Under visible-light irradiation, both FePc and HCNT can be excited to generate electron–hole pairs (Equations (7) and (8)). Interfacial coupling may promote the separation and effective utilization of photogenerated carriers by enabling the recombination of relatively low-energy carriers at the interface while preserving carriers with stronger redox capability. Surface nitrogen vacancies play an important role in this process. As evidenced by the reduced PL intensity, enhanced photocurrent response, and increased charge extraction of HCNT relative to CNT, nitrogen-vacancy engineering suppresses carrier recombination and improves photogenerated-charge separation and utilization. Consequently, more photogenerated carriers remain available for subsequent surface redox reactions. The electrons retained on the catalyst surface can react with dissolved oxygen to generate ∙O_2_^−^ (Equation (11)), which agrees with the dominant contribution of ∙O_2_^−^ revealed by scavenger experiments. In parallel, photogenerated holes can directly oxidize OTC or participate in other oxidative pathways. The generated ∙O_2_^−^ may further undergo secondary reactions that contribute to ^1^O_2_ formation (Equation (12)). Dissolved O_2_ is expected to be the main oxygen source, while O_2_ generated through H_2_O_2_ decomposition may provide an additional contribution (Equation (10)).

In addition to the photocatalytic pathway, the component dependent experiments described in Section 3.3 demonstrate that FePc serves as the primary center for H_2_O_2_ activation, whereas HCNT functions as a defect-engineered tubular photocatalytic platform for photogenerated-carrier generation and ROS formation. The Fe centers in FePc participate in Fe(II)/Fe(III)-mediated Fenton reactions, promoting H_2_O_2_ activation and the generation of ∙OH (Equation (9)). The interfacial interaction between FePc and HCNT facilitate the utilization of photogenerated carriers and help maintain the catalytic activity of Fe sites according to Equation (13). Nevertheless, because the dynamic evolution of Fe oxidation states was not directly monitored, this regeneration pathway is considered a possible contribution rather than a conclusively demonstrated mechanism [42]. The proposed reactions can be expressed as follows:

(7) $$g\text{-}C_{3}N_{4}\;+\;h\nu \;\to \;g\text{-}C_{3}N_{4}\;+\;h^{+}\;+\;e^{-}$$

(8) $${\mathrm{Fe}}^{2+}\mathrm{Pc}+h\nu \;\to \;{\mathrm{Fe}}^{3+}\mathrm{Pc}+e^{-}$$

(9) $$e^{-}+{\;H}_{2}O_{2}\;\to \;\cdot \mathrm{OH}+{\mathrm{OH}}^{-}$$

(10) $$H_{2}O_{2}\;\to \;H_{2}O+O_{2}$$

(11) $$O_{2}+e^{-}\;\to \;\cdot O_{2^{-}}$$

(12) ·O2−+h+ → O21

(13) $${\mathrm{Fe}}^{3+}\mathrm{Pc}+e^{-}\;\to \;{\mathrm{Fe}}^{2+}\mathrm{Pc}$$

Overall, the enhanced photo-Fenton activity of FePc/HCNT originates from the complementary functions of molecular Fe sites, nitrogen-vacancy-engineered HCNT, and their interfacial coupling. FePc provides the primary catalytic centers for H_2_O_2_ activation, whereas HCNT promotes light harvesting, photogenerated-carrier production, and ROS formation. Surface nitrogen vacancies suppress carrier recombination and enhance charge separation and utilization, thereby increasing the availability of photogenerated carriers for oxygen reduction and other surface reactions. Through the synergistic coupling of FePc-mediated H_2_O_2_ activation and HCNT-assisted photocatalytic oxidation, multiple reactive species, including ∙O_2_^−^, h^+^, ^1^O_2_, and ∙OH, are generated and collectively promote efficient OTC degradation.

### 3.6. OTC Degradation Intermediates and Toxicity Analysis

**Figure 7:**
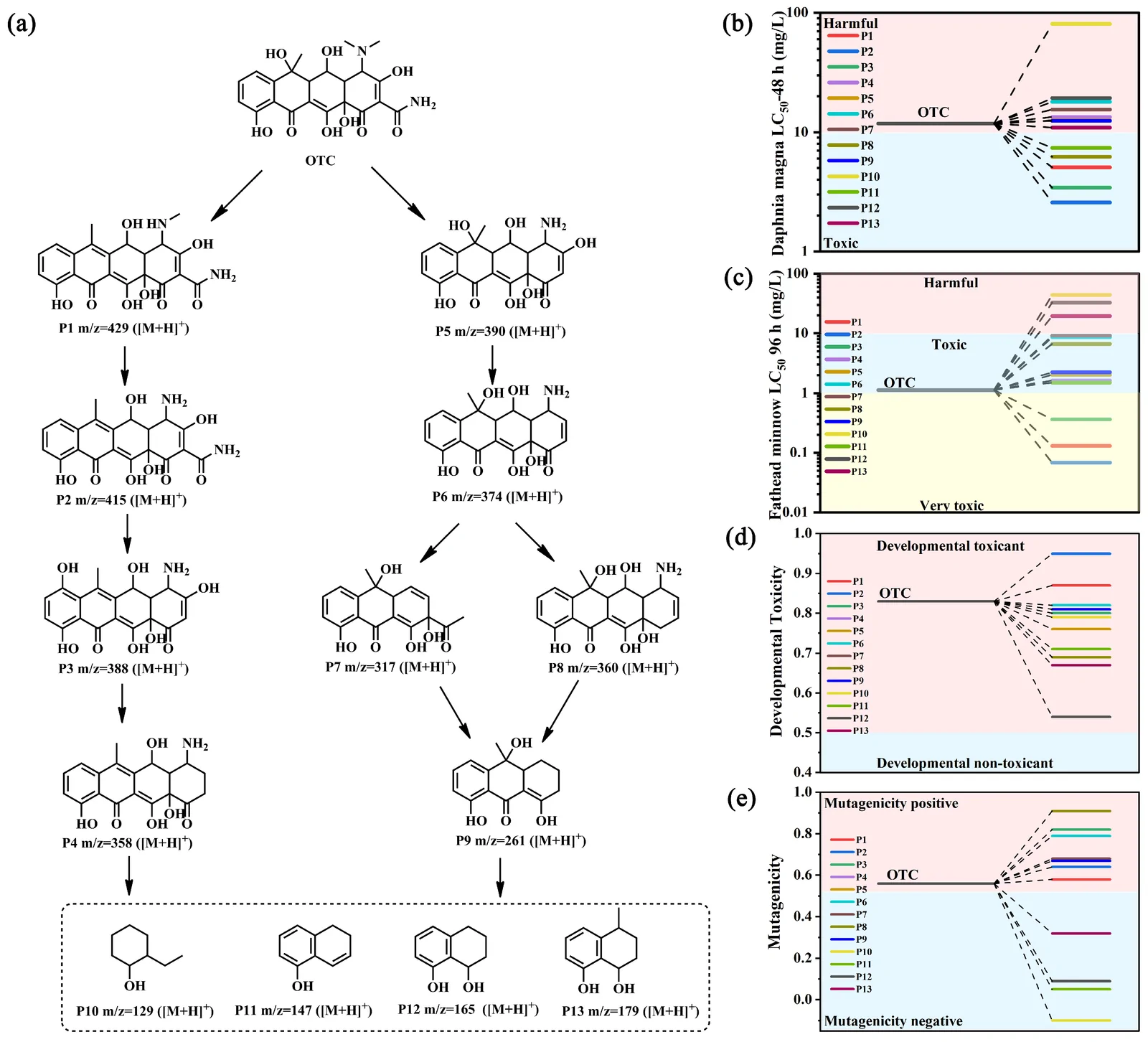
(**a**) Proposed OTC degradation pathways in the FePc/HCNT/Vis/H_2_O_2_ system (20 min irradiation) by LC-MS; (**b**) 48 h-LC_50_ for large flea, (**c**) 96 h-LC_50_ for blackhead minnow. (**d**) Developmental toxicity. (**e**) Mutagenicity of OTC and its intermediates predicted by T.E.S.T. software (version 5.1).

The main degradation intermediates formed in the OTC solution after 20 min of the photo-Fenton reaction were analyzed by liquid chromatography–mass spectrometry (LC-MS). Representative mass spectra are provided in Figure S16, while the proposed structures and measured *m*/*z* values of the detected intermediates are summarized in Table S3. Based on these data, the proposed degradation pathways of OTC are drawn in Figure 7a. Two main routes are involved in OTC degradation. In the first route, OTC undergoes dehydroxylation and deamination to form P1 (*m*/*z* = 429) [59,60]. P1 then loses a methyl group to give P2 (*m*/*z* = 415) [59,61,62]. P2 is further converted to P3 (*m*/*z* = 388) [61] via deamidation and hydroxylation, and P3 finally turns into P4 (*m*/*z* = 358) after another dehydroxylation step. The second route starts with the removal of the amide group from OTC to produce P5 (*m*/*z* = 390) [63]. P2 is transformed to P6 (*m*/*z* = 374) by dehydroxylation. P6 then undergoes deamination and ring opening to form P7 (*m*/*z* = 317) [61]. At the same time, P6 can also lose a carbonyl group to yield P8 (*m*/*z* = 360) [63]. Both P7 and P8 are further oxidized to the same intermediate P9 (*m*/*z* = 261) [60]. As the oxidation continues, P4 and P9 break down into smaller fragments, including P10 (*m*/*z* = 129), P11 (*m*/*z* = 147), P12 (*m*/*z* = 165) [59], and a second product originally also labeled as P12 in the text, here more clearly distinguished as P13 (*m*/*z* = 179) [59].

Evaluating the toxicity of degradation intermediates is necessary to understand the potential environmental risks of OTC during its breakdown. In this work, four toxicity indicators (48 h-LC_50_ for large flea, 96 h-LC_50_ for blackhead minnow, developmental toxicity, and mutagenicity) were predicted for OTC and its main intermediates using the T.E.S.T. software (version 5.1), which is based on a quantitative structure–activity relationship (QSAR) model [64]. According to the Globally Harmonized System of Classification and Labelling of Chemicals (GHS) [65], the toxicity of each intermediate was classified by its LC_50_ value: LC_50_ ≤ 1 mg L^−1^ is considered very toxic; 1 mg L^−1^ < LC_50_ ≤ 10 mg L^−1^ is considered toxic; and LC_50_ > 10 mg L^−1^ is considered harmful. The LC_50_-48 h and LC_50_-96 h values refer to the concentration (mg L^−1^) of the test chemical in water that causes 50% mortality in the tested organisms after 48 and 96 h, respectively. As shown in Figure 7b, half of the detected intermediates have LC_50_-48 h values higher than that of OTC, meaning most of them are less toxic to large fleas than the parent compound, and most products fall into the low-toxicity category. Figure 6c presents the LC_50_-96 h results for blackhead minnow. Except for P1, P2, and P3, the other ten intermediates show LC_50_-96 h values higher than that of OTC, indicating lower toxicity than the original compound. For developmental toxicity (Figure 6d), all intermediates except P1 and P2 exhibit lower values than OTC, demonstrating the effective detoxification ability of this system. Regarding mutagenicity (Figure 6e), although most intermediates show higher values than OTC, the final products P10–P13 are less toxic and show negative mutagenicity. These results indicate that the proposed system has good potential for treating OTC-contaminated wastewater. Under continuous visible light (Figure S17), the TOC removal rate of OTC over FePc/HCNT increased steadily, showing sustained mineralization ability of the photo-Fenton system. The TOC removal reached 14% after 4 h of illumination.

### 3.7. Practical Application Potential

**Figure 8:**
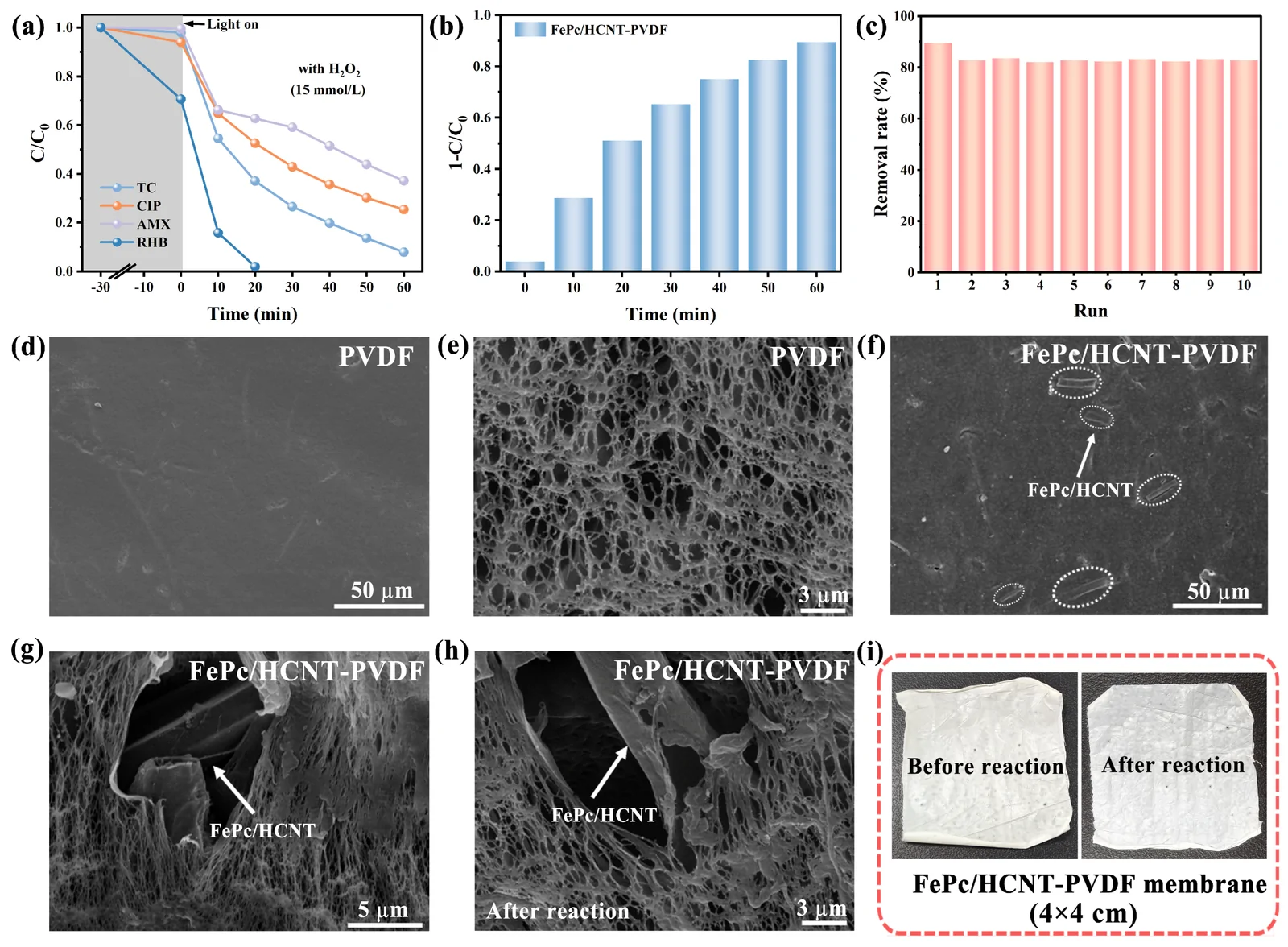
(**a**) Degradation curves of various pollutants over FePc/HCNT in the photo-Fenton system. (**b**) Photocatalytic degradation of OTC over FePc/HCNT-PVDF membrane. (**c**) Recycling efficiency of FePc/HCNT-PVDF membrane for photo-Fenton degradation of OTC. (**d**,**e**) SEM images of bare PVDF membrane. (**f**,**g**) SEM images of FePc/HCNT-PVDF membrane. (**h**) SEM images of FePc/HCNT-PVDF membrane after cycling. (**i**) Digital optical images of FePc/HCNT-PVDF membrane before and after use.

The applicability of FePc/HCNT toward different classes of refractory organic pollutants was evaluated using rhodamine B (RhB), tetracycline, amoxicillin, and ciprofloxacin as representative contaminants. As shown in Figure 8a, all selected pollutants underwent appreciable degradation within 60 min at 25 °C. The corresponding removal efficiencies reached 100% for RhB, 94% for tetracycline, 63% for amoxicillin, and 75% for ciprofloxacin. Although the degradation efficiencies varied with the molecular structures and reactivities of the pollutants, these results demonstrate that FePc/HCNT is capable of treating structurally diverse organic contaminants rather than being limited to OTC alone.

Despite its high catalytic activity, the direct use of powdered FePc/HCNT may present practical limitations, including difficult recovery, catalyst loss, and potential secondary contamination [66]. To improve its recyclability and operational feasibility, FePc/HCNT was immobilized within a polyvinylidene fluoride (PVDF) membrane, and the resulting FePc/HCNT-PVDF membrane was evaluated for OTC degradation. As shown in Figure 8b, the FePc/HCNT-PVDF membrane achieved approximately 90% OTC degradation within 60 min, demonstrating its appreciable activity toward OTC degradation.

The reusability of the FePc/HCNT-PVDF membrane was further assessed through repeated degradation experiments (Figure 8c). The membrane maintained stable OTC removal performance during consecutive cycles, demonstrating its favorable operational stability. SEM images acquired before and after use (Figure 8d–g) show that the micrometer-scale tubular FePc/HCNT structures remained exposed on the surface of the porous PVDF matrix. This surface-exposed configuration differs from the common encapsulation of nanoscale catalyst particles within polymer matrices, which may hinder reactant diffusion and reduce the accessibility of catalytic sites [67,68,69]. The tubular morphology of FePc/HCNT therefore helps preserve accessible catalytic interfaces during membrane immobilization and repeated operation, as schematically illustrated in Figure 8h. No obvious morphological damage or structural collapse was observed after the degradation experiments, confirming the good durability of the membrane. Moreover, with a low FePc/HCNT loading of approximately 0.1 mg cm^−2^, the membrane still achieved effective pollutant removal, highlighting its potential for reusable visible-light-assisted wastewater treatment. Digital photographs of the membrane before and after use are shown in Figure 8i.

## 4. Conclusions

In summary, a FePc/HCNT molecular heterojunction was constructed by integrating tubular architecture engineering, surface nitrogen-vacancy regulation, molecular FePc catalysis, and membrane-based immobilization toward efficient and reusable photo-Fenton degradation of antibiotics. The unique tubular g-C_3_N_4_ framework provided accessible catalytic interfaces and favorable mass-transfer pathways, while surface N_v_ regulation effectively tuned the electronic structure of HCNT and enhanced the separation and utilization of photogenerated carriers. Systematic component-dependent investigations further confirmed the complementary functions of FePc and HCNT in the photo-Fenton process, where FePc contributed to H_2_O_2_ activation through molecular Fe catalytic sites, while nitrogen-vacancy-rich HCNT functioned as a defect-engineered tubular photocatalytic platform, contributing to photogenerated-carrier utilization and ROS formation. The integration of these two components promoted the cooperative utilization of light energy and H_2_O_2_, resulting in enhanced antibiotic degradation performance. Moreover, the micrometer-scale tubular morphology of HCNT facilitated membrane immobilization by maintaining surface-exposed catalytic interfaces and reducing catalyst encapsulation within the polymer matrix, which contributed to the excellent reusability of the photocatalytic system. This work contributes to a better understanding of the structure–activity relationship in FePc/g-C_3_N_4_ photo-Fenton systems and supports the further development of reusable immobilized catalysts.

## Data Availability

Data available on request from the authors.

## Supplementary Materials

The following supporting information can be downloaded at: https://www.mdpi.com/article/10.3390/molecules31183229/s1, Figure S1: Image of the experimental setup for evaluating the catalytic performance of the FePc/HCNT-PVDF membrane; Figure S2: Digital optical images of CNT, HCNT and FePc/HCNT-X; Figure S3: The SEM image of the CNT precursor; Figure S4: The SEM images of (a) CN, (b) CNS; Figure S5: Adsorption performance of FePc, CNT, HCNT, and FePc/HCNT-5 toward OTC; Figure S6: Photo-Fenton degradation performance of OTC over the FePc/HCNT-5 heterojunction and the corresponding individual-component control samples; Figure S7: Fe leaching concentration of FePc/HCNT during the photo-Fenton reaction determined by inductively coupled plasma optical emission spectrometry (ICP-OES, Agilent 5800); Figure S8: EPR spectra of FePc/HCNT-5 samples obtained after different calcination times under an H_2_/Ar reducing atmosphere; Figure S9: OTC degradation performance of HCNT samples synthesized at different temperatures and their corresponding FePc-based heterojunctions in the photo-Fenton system; Figure S10: Ultraviolet-visible diffuse reflection spectroscopy (DRS) of CN, CNT, HCNT samples; Figure S11: Band gaps of (a–c) CNT, HCNT and FePc; Figure S12: Valence band XPS spectra of (a–c) CNT, HCNT and FePc; Figure S13: Band structures of CNT, HCNT and FePc; Figure S14: Electron decay constants (*τ*) of CNT, HCNT and FePc/HCNT estimated from TPV spectra; Figure S15: Number of effective charges (neff) of CNT, HCNT and FePc/HCNT estimated from TPV spectra; Figure S16: Mass spectra of OTC intermediates at different retention times; Figure S17: TOC removal rate of FePC/HCNT catalyst for OTC degradation. Table S1: BET data of catalysts; Table S2: Transient photovoltage parameters of the samples; Table S3: Proposed degradation intermediates of OTC identified by HPLC-MS.
