# Supplementary material for: Nitrogen Vacancy Rich Tubular g-C3N4 Modified with FePc for Efficient and Reusable Photo-Fenton Degradation of Oxytetracycline
Source: Molecules. 2026 Sep 12;31(18):3229. doi: 10.3390/molecules31183229 (PMC13609133; doi:10.3390/molecules31183229)
Supplement: Supplementary file 1 [file molecules-31-03229-s001.zip › molecules-4510316-supplementary.pdf]

Supplementary Information

Nitrogen Vacancy-Mediated FePc Molecular Heterojunction on Tubular g-C_3N_4 for Efficient Photo-Fenton Degradation of Oxytetracycline

Xinyi Yang[a], Yuxin Tang[a], Fei Qi[b], Zhihan Xue[c], Huiying Zhang[b], Zhaohai Ni[b], Bo Feng[b,*], Ziyang Yue[c,*], Guangbo Che[b,*]

[a]*College of Safety Science and Engineering, Liaoning Technical University, Huludao 125105, P. R. China.*

[b]*Jilin Provincial Key Laboratory of Western Jilin's Clean Energy, Baicheng Normal University, Baicheng 137000, P. R. China.*

[c]*College of Chemistry, Changchun Normal University, Changchun, 130032, Jilin, P.R. China.*

[d]*College of Science, Liaoning Technical University, Fuxin 123000, P. R. China.*

***Corresponding authors:**

Dr. Bo Feng: fengbo@bcnu.edu.cn

Dr. Ziyang Yue: 13591995463@163.com

Prof. Guangbo Che: guangboche@bcnu.edu.cn

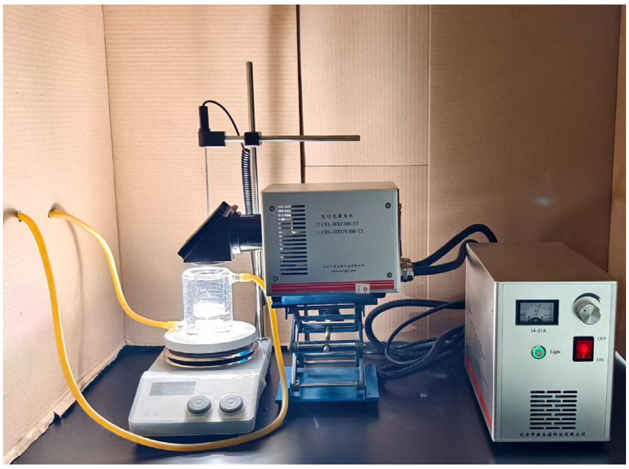

Figure S1. Image of the experimental setup for evaluating the catalytic performance of the FePc/HCNT-PVDF membrane.

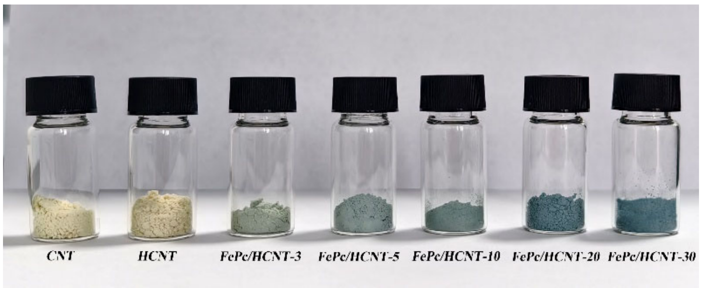

Figure S2. Digital optical images of CNT, HCNT and FePc/HCNT-X.

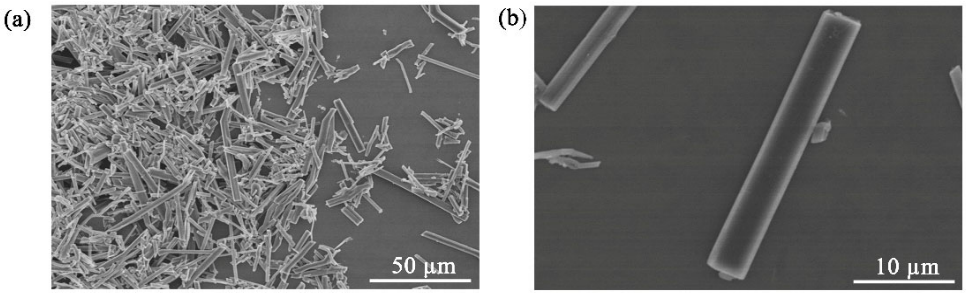

Figure S3. The SEM image of the CNT precursor.

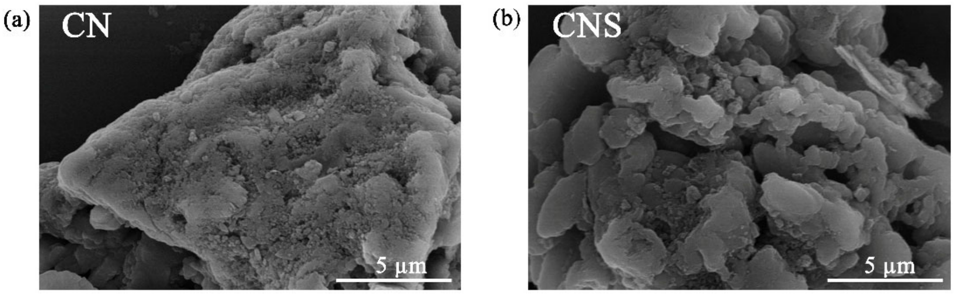

Figure S4. The SEM images of (a) CN, (b) CNS.

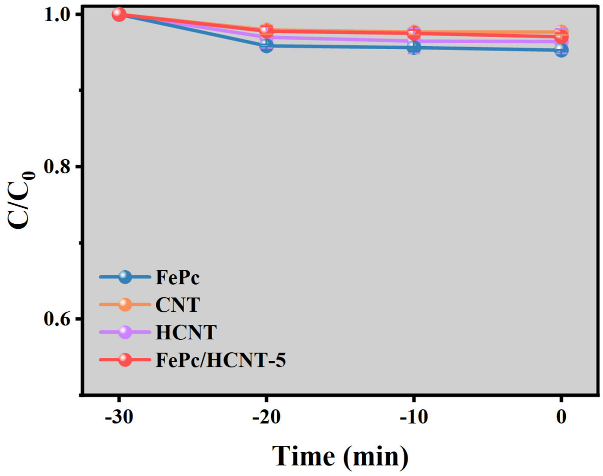

Figure S5. Adsorption performance of FePc, CNT, HCNT, and FePc/HCNT-5 toward OTC.

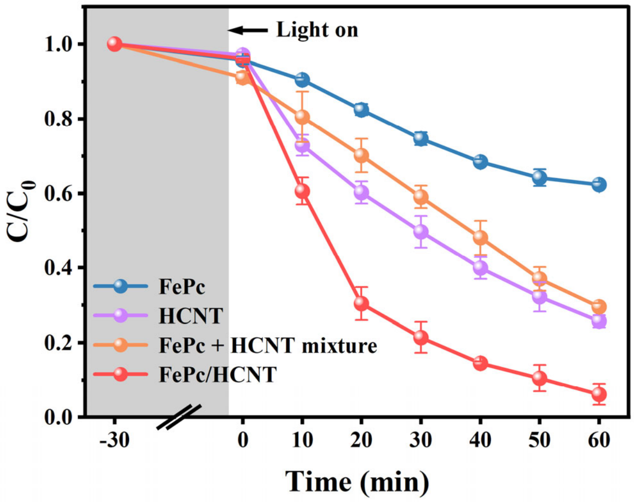

Figure S6. Photo-Fenton degradation performance of OTC over the FePc/HCNT-5 heterojunction and the corresponding individual-component control samples.

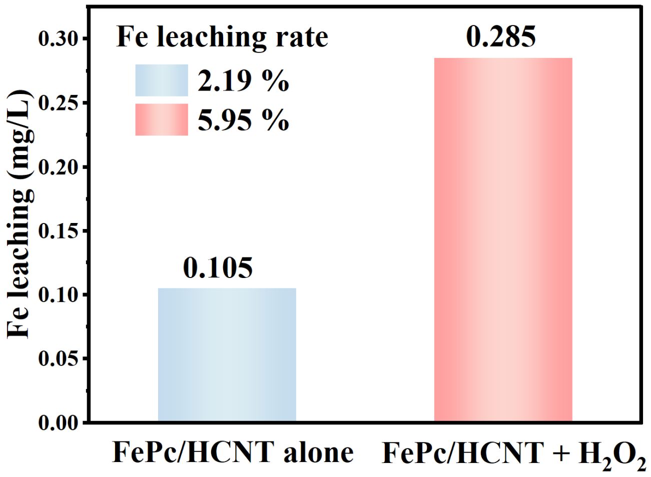

Figure S7. Fe leaching concentration of FePc/HCNT during the photo-Fenton reaction determined by inductively coupled plasma optical emission spectrometry (ICP-OES, Agilent 5800).

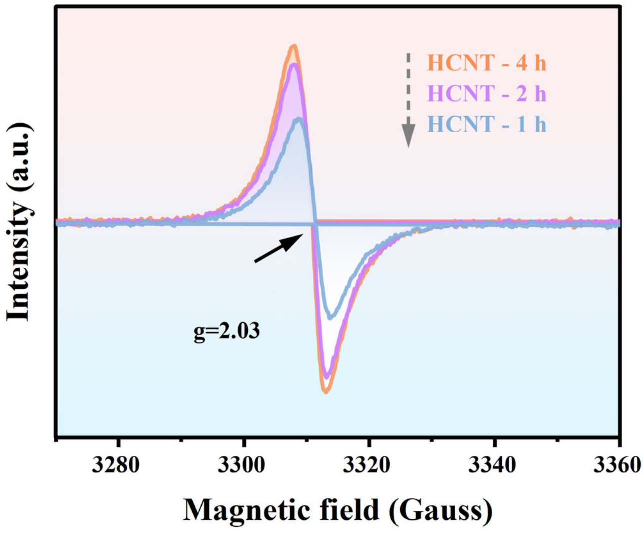

Figure S8. EPR spectra of FePc/HCNT-5 samples obtained after different calcination times under an H_2/Ar reducing atmosphere.

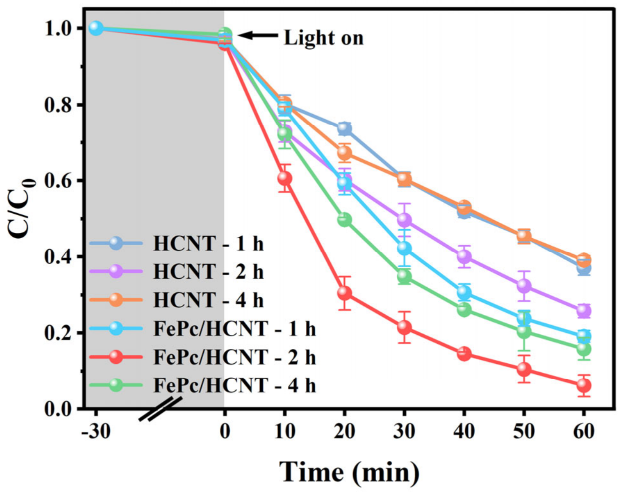

Figure S9. OTC degradation performance of HCNT samples synthesized at different temperatures and their corresponding FePc-based heterojunctions in the photo-Fenton system.

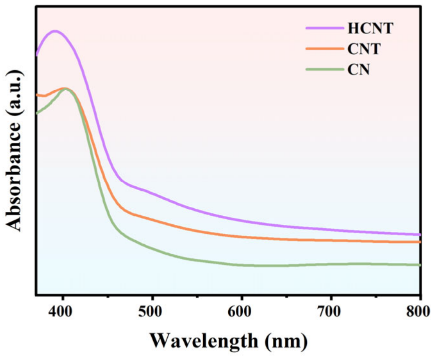

Figure S10. Ultraviolet-visible diffuse reflection spectroscopy (DRS) of CN, CNT, HCNT samples.

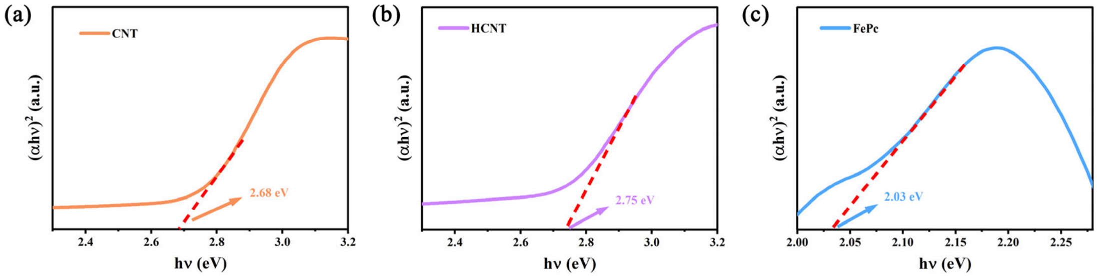

Figure S11. Band gaps of (a-c) CNT, HCNT and FePc.

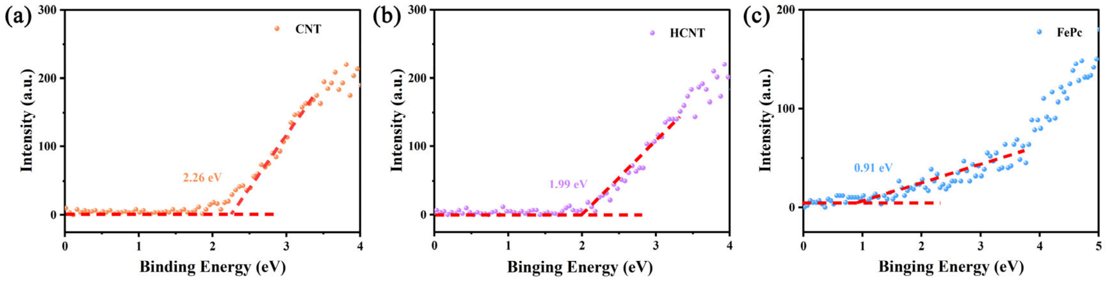

Figure S12. Valence band XPS spectra of (a-c) CNT, HCNT and FePc.

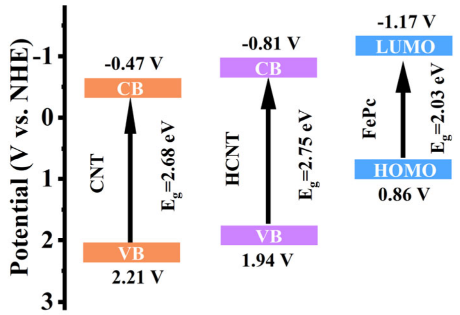

Figure S13. Band structures of CNT, HCNT and FePc.

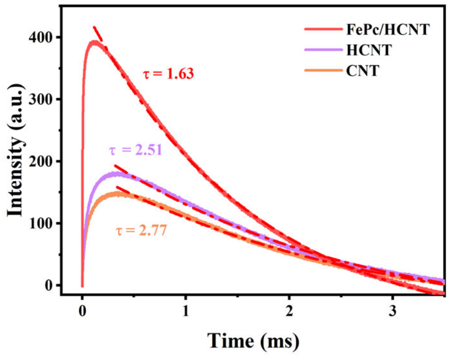

Figure S14. Electron decay constants (τ) of CNT, HCNT and FePc/HCNT estimated from TPV spectra.

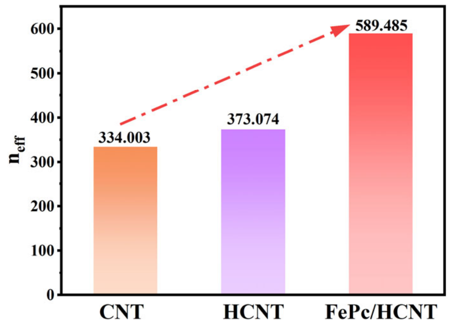

Figure S15. Number of effective charges (neff) of CNT, HCNT and FePc/HCNT estimated from TPV spectra.

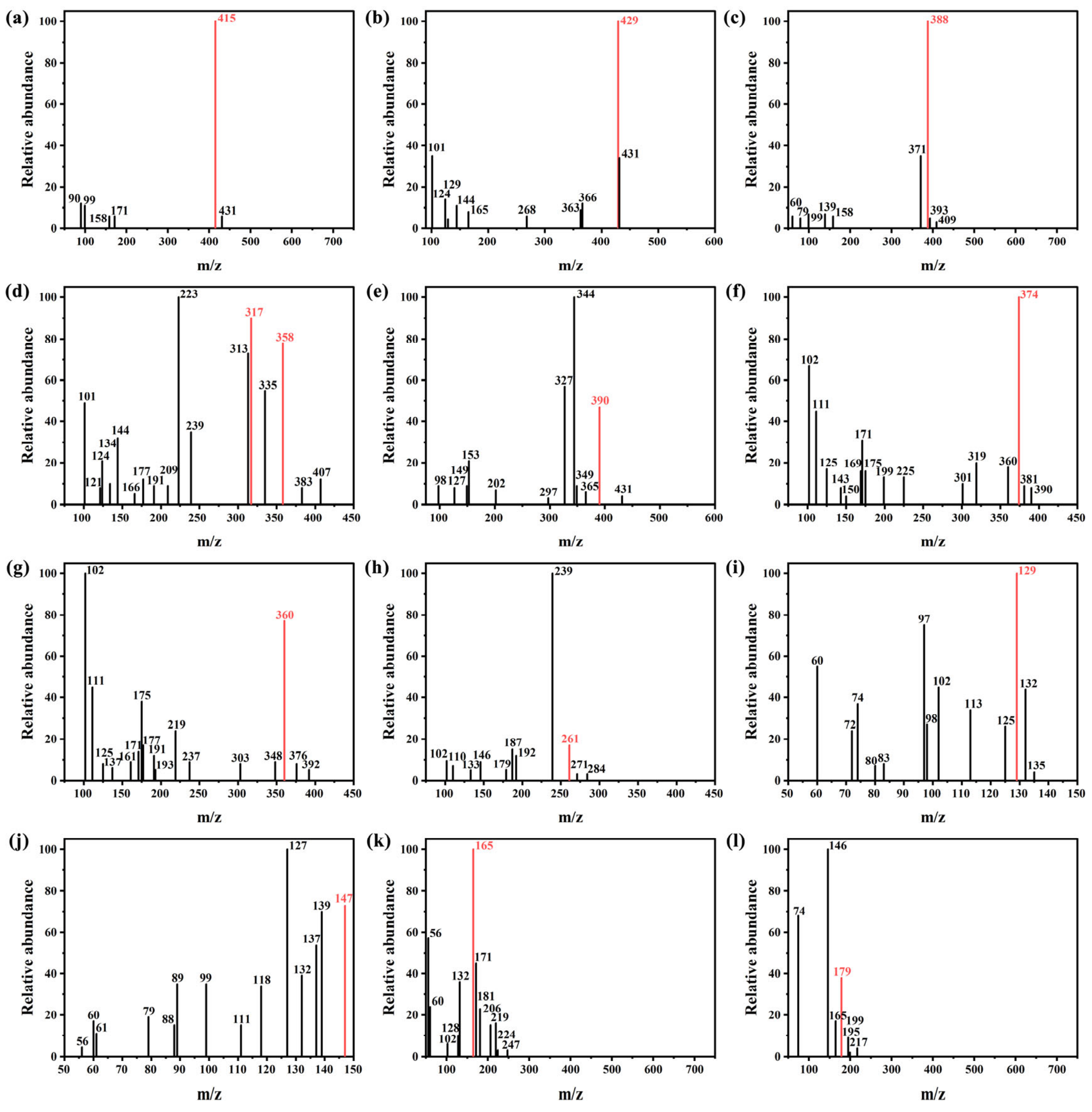

Figure S16. Mass spectra of OTC intermediates at different retention times.

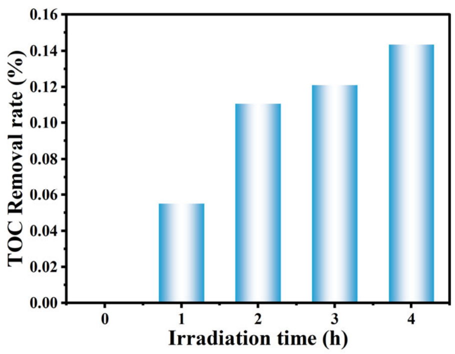

Figure S17. TOC removal rate of FePC/HCNT catalyst for OTC degradation.

Table S1. BET data of catalysts.

**Catalyst**	**S_{BET} (m^2/g)**
CNT	13.3861
HCNT	50.3815
FePc/HCNT-3	31.9441
FePc/HCNT-5	24.8377
FePc/HCNT-10	17.7208
FePc/HCNT-20	27.9659
FePc/HCNT-30	38.1218

Table S2. Transient photovoltage parameters of the samples.

**Photocatalyst**	**t_{max}**	**A**	**τ**	**n_{eff}**
CNT	0.33063	40.99287	2.77	334.003
HCNT	0.30302	44.96184	2.51	373.074
FePc/HCNT	0.13688	49.37660	1.63	589.485

Table S3. Proposed degradation intermediates of OTC identified by HPLC-MS.

No.	Fomula	Chemical Structure	m/z of $[M+H]^+$
P1	$C_{21}H_{20}N_2O_8$	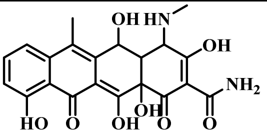	429
P2	$C_{20}H_{18}N_2O_8$	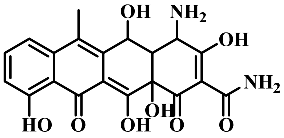	415
P3	$C_{19}H_{17}NO_8$	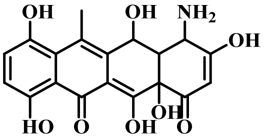	388
P4	$C_{19}H_{19}NO_6$	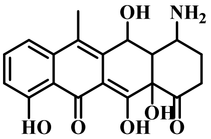	358
P5	$C_{19}H_{19}NO_8$	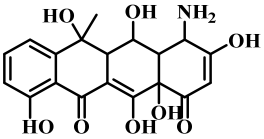	390

No.	Fomula	Chemical Structure	m/z of $[M+H]^+$
P6	$C_{19}H_{19}NO_7$	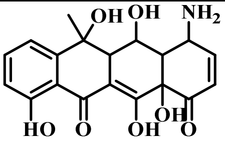	374
P7	$C_{17}H_{16}O_6$	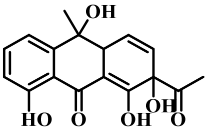	317
P8	$C_{19}H_{21}NO_6$	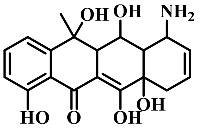	360
P9	$C_{15}H_{16}O_4$	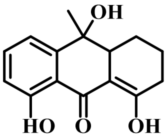	261
P10	$C_8H_{16}O$	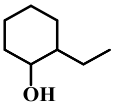	129
P11	$C_{10}H_{10}O$	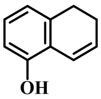	147
P12	$C_{10}H_{12}O_2$	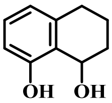	165
P13	$C_{11}H_{14}O_2$	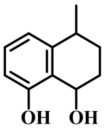	179
